# ZFP36-mediated mRNA decay regulates metabolism

**DOI:** 10.1016/j.celrep.2023.112411

**Published:** 2023-04-21

**Authors:** Andrew C. Cicchetto, Elsie C. Jacobson, Hannah Sunshine, Blake R. Wilde, Abigail S. Krall, Kelsey E. Jarrett, Leslie Sedgeman, Martin Turner, Kathrin Plath, M. Luisa Iruela-Arispe, Thomas Q. de Aguiar Vallim, Heather R. Christofk

**Affiliations:** 1 Department of Biological Chemistry, University of California, Los Angeles (UCLA), Los Angeles, CA, USA; 2 Department of Cell and Developmental Biology, Feinberg School of Medicine, Northwestern University, Chicago, IL, USA; 3 Department of Medicine, Division of Cardiology, UCLA, Los Angeles, CA, USA; 4 Immunology Programme, The Babraham Institute, Cambridge, UK; 5 Eli and Edythe Broad Center of Regenerative Medicine and Stem Cell Research, UCLA, Los Angeles, CA, USA; 6 Molecular Biology Institute, UCLA, Los Angeles, CA, USA; 7 Jonsson Comprehensive Cancer Center, UCLA, Los Angeles, CA, USA; 8 Lead contact

## Abstract

Cellular metabolism is tightly regulated by growth factor signaling, which promotes metabolic rewiring to support growth and proliferation. While growth factor-induced transcriptional and post-translational modes of metabolic regulation have been well defined, whether post-transcriptional mechanisms impacting mRNA stability regulate this process is less clear. Here, we present the ZFP36/L1/L2 family of RNA-binding proteins and mRNA decay factors as key drivers of metabolic regulation downstream of acute growth factor signaling. We quantitatively catalog metabolic enzyme and nutrient transporter mRNAs directly bound by ZFP36 following growth factor stimulation—many of which encode rate-limiting steps in metabolic pathways. Further, we show that ZFP36 directly promotes the mRNA decay of Enolase 2 (Eno2), altering Eno2 protein expression and enzymatic activity, and provide evidence of a ZFP36/Eno2 axis during VEGF-stimulated developmental retinal angiogenesis. Thus, ZFP36-mediated mRNA decay serves as an important mode of metabolic regulation downstream of growth factor signaling within dynamic cell and tissue states.

## In brief

Growth factor signaling rapidly reprograms cellular metabolism. Cicchetto et al. show that the RNA-binding protein ZFP36, induced by growth factor signaling, acutely regulates metabolic gene expression through altered mRNA stability.

## Graphical abstract

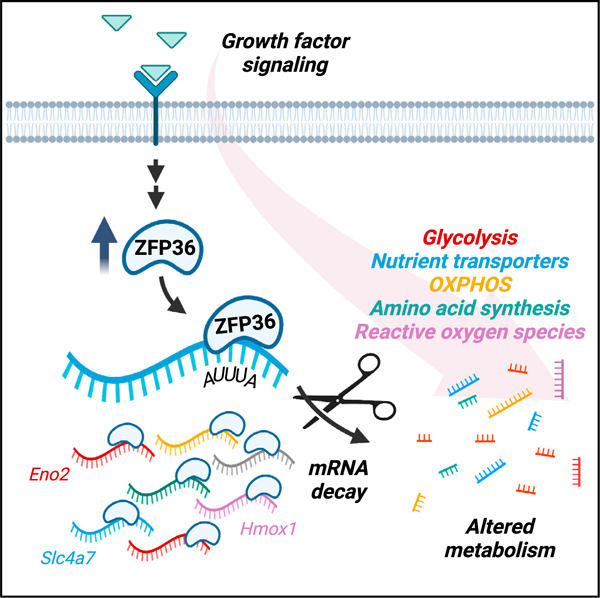

## INTRODUCTION

Cellular responses to extrinsic cues often require a coordinated change in metabolism. Downstream of growth factor- or cytokine-induced signaling pathway activation, metabolism is regulated at the transcriptional level by transcription factors such as HIF, MYC, and SREBP.^1,2^ Metabolism is also regulated at the post-translational level via phosphorylation of metabolic enzymes, such as AKT phosphorylation of ACLY to promote acetyl-coenzyme A (CoA) production, PANK2/4 to promote CoA synthesis, and NADK to stimulate NADP+ production.^3–6^ However, less is known about how metabolism is regulated post-transcriptionally at the level of mRNA stability. This is notable since some RNA-binding proteins are immediate-early genes that can quickly alter mRNA stability and thereby impact overall gene expression in response to a stimulus.^7–9^

The ZFP36 family of RNA-binding proteins is induced by a variety of external stimuli and plays a role in mediating adaptive cellular processes.^10^ These proteins, comprised of three family members—ZFP36, ZFP36L1, and ZFP36L2—perform mRNA decay by facilitating poly-A tail deadenylation, the rate-limiting step of mRNA degradation.^11,12^ ZFP36 proteins contain a highly conserved CCCH tandem zinc finger domain endowing them the capacity to bind AU-rich elements (AREs) in the 3′ UTR of target mRNA transcripts, which in turn serves as a scaffold for the CCR4/CAF1/NOT1 deadenylase complex.^13^ A large proportion of short-lived inducible transcripts possess AREs.^7,14^ Moreover, *in silico* analyses estimate that nearly 10% of the eukaryotic genome encode mRNAs harboring these elements suggesting, at least in biochemical terms, a theoretical susceptibility to ZFP36-mediated decay.^15^ Given that metabolic enzyme gene transcripts are among the mRNAs containing AREs, the extent to which ZFP36-mediated mRNA decay influences cellular metabolism merits investigation.^7^

Here, we show that acute growth factor signaling is a potent activator of ZFP36 expression and mRNA decay activity. We quantitatively catalog mRNAs directly bound by ZFP36 and identify metabolic enzymes and nutrient transporters as bona fide targets of ZFP36. We show that protein expression of one identified ZFP36-bound mRNA, the glycolytic enzyme Enolase 2, is highly regulated by ZFP36-mediated mRNA decay. Lastly, we provide evidence that ZFP36 regulation of Enolase 2 (ENO2) occurs during neonatal development and contributes to VEGF-stimulated retinal angiogenesis *in vivo*.

## RESULTS

### Acute growth factor signaling induces ZFP36 family member expression *in vitro* and *in vivo*

**Figure 1.**
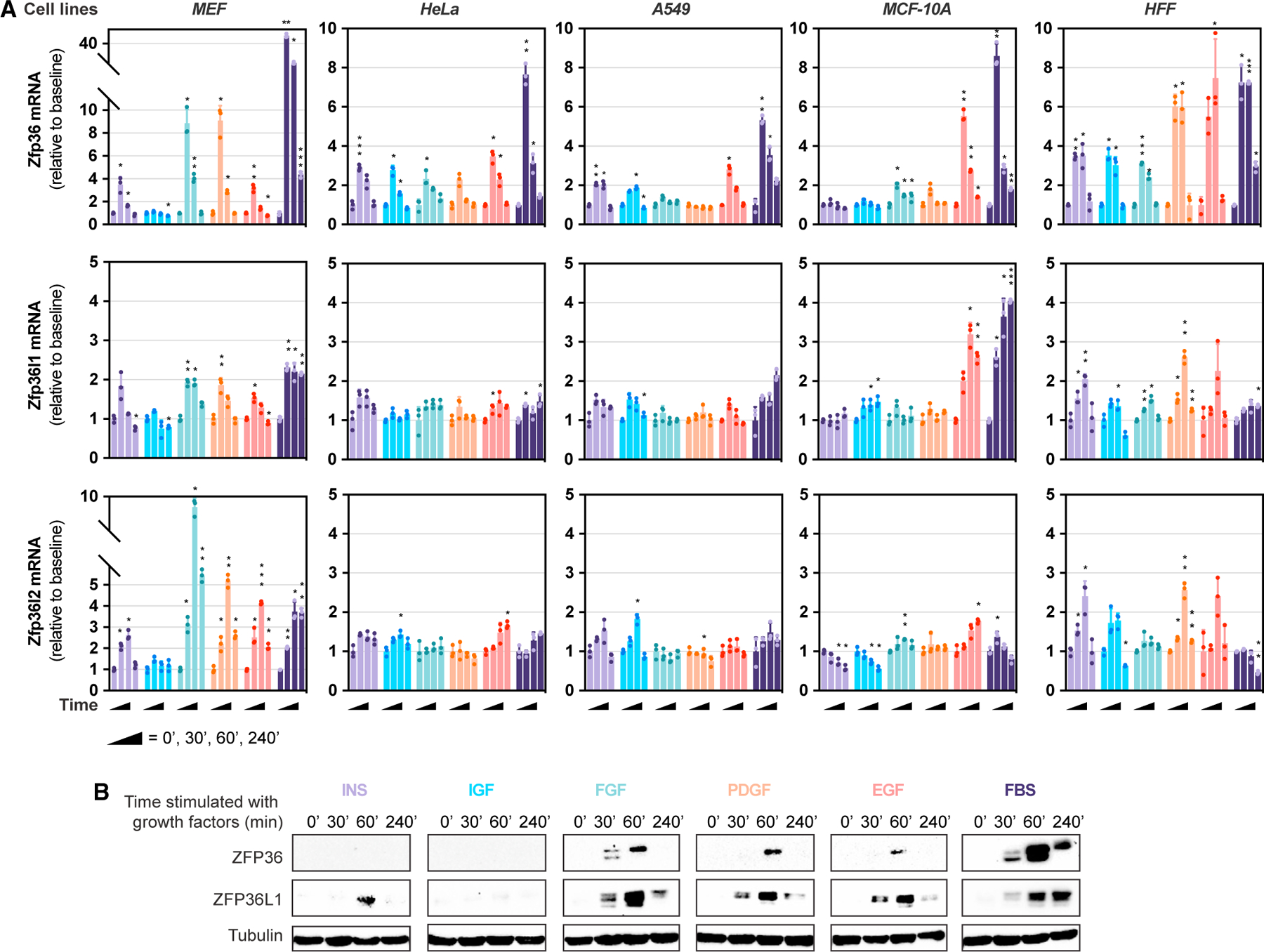
Acute growth factor signaling induces ZFP36 family member expression (A and B) Relative Zfp36/l1/l2 transcript levels (A) or immunoblotting of MEF lysates for ZFP36, ZFP36L1, and tubulin (B) in response to growth factor stimulation (insulin, 1 μg/mL; Igf-1, 100 ng/mL; βFGF, 10 ng/mL; PDGF-BB, 10 ng/mL; EGF, 10 ng/mL; or 10% FBS) for 0, 30, 60, or 240 min following overnight serum deprivation. Commercially available antibodies for ZFP36L2 are not available for mouse cells, and therefore ZFP36L2 is absent from immunoblotting analyses. All experiments were performed with biological replicates. Error bars denote SD (n = 3). *p < 0.05; **p < 0.01; ***p < 0.001. A two-way ANOVA Dunnett’s multiple comparisons test was performed for (A).

To determine the temporality of ZFP36 family member induction in response to growth factor stimulation, we examined the kinetics of Zfp36, Zfp36l1, and Zfp36l2 mRNA expression post-acute exposure of multiple cell lines to various stimuli. We conducted this analysis on a small panel of cultured cell lines—mouse embryonic fibroblasts (MEFs), HeLa, A549, MCF-10A, and human foreskin fibroblasts (HFF-1)—each stimulated with an array of individual growth factors (insulin, IGF-1, bFGF, PDGF-BB, or EGF) at near-physiological concentrations or with serum (fetal bovine serum [FBS]), and RNA was harvested at multiple time points post-stimulation (Figure 1A). We also assessed temporality of ZFP36 induction in the hepatocellular carcinoma (HCC) cell response to serum, bone marrow-derived macrophage (BMDM) response to lipopolysaccharide (LPS), and human umbilical vein endothelial cell (HUVEC) response to VEGF (Figure S1A). Through our scaled approach, interrogating multiple time points—30, 60, and 240 min—post-stimulation, we captured the dynamic changes in Zfp36 family member mRNA expression during the initial cellular transition from a resting to growing cell state. Further, Zfp36 mRNA in particular appeared to be the most acutely responsive to growth factor signaling, peaking within 30 min. A minority of cell lines responded to growth signals by downregulating Zfp36l2 mRNA specifically, as evident in serum-stimulated HFF-1 cells and insulin/IGF-1-stimulated MCF-10A cells. Importantly, not all individual growth factor stimuli induced ZFP36 family member expression across the cell lines tested, perhaps due to the cell lines’ epigenetic and genetic background or display of cognate growth factor receptors on the cell surface. However, serum stimulation increased Zfp36 levels across all cell lines tested, with MEFs exhibiting the largest dynamic range (Figure 1A). The relative potency of serum-induced ZFP36 and ZFP36L1 protein expression was confirmed in MEFs (Figures 1B and S1B). Consistent with previous reports, rapid and robust induction of ZFP36 and ZFP36L1 protein expression was also observed in livers harvested from mice 15-min post-insulin stimulation (Figures S1C and S1D).^16^ Together, these findings couple growth factor signaling with ZFP36 family member expression on a stimulus- and cell type-specific basis *in vitro* and *in vivo*.

### ZFP36 proteins are immediate-early genes downstream of MAPK and mTORC1/2 signaling

Given the rapid onset of ZFP36 family member expression in response to growth factor stimulation, we next sought to define the mechanism by which ZFP36 proteins are induced. To this end, we examined how inhibitors of kinases downstream of RTK signaling (MAPK and mTORC1/2) as well as inhibitors of transcription or translation impact ZFP36/L1/L2 induction by serum stimulation. We found that ZFP36/L1/L2 induction is abrogated by MAPK inhibition using a combined Mek and p38 inhibitor cocktail (MAPKi) (Figures S2A–S2D). Torin, a potent and selective mTOR kinase inhibitor, had no effect on Zfp36/l1/l2 mRNA induction in the presence of FBS but reduced protein expression (Figure S2E–S2H). Similarly, blocking translational elongation with cycloheximide (CHX) restricts ZFP36/L1 protein expression without affecting mRNA levels, suggesting a lack of requirement for cofactor expression to facilitate Zfp36 mRNA onset and that nascent protein synthesis underlies ZFP36 family member expression (Figures S2I–S2L). Interestingly, we observed that CHX alone causes a slight but significant induction of Zfp36 and Zfp36l1 mRNA, though this was not additive with FBS cotreatments, contrary to previously published reports (Figures S2J and S2K).^16^ Further, a CHX-chase assay revealed that the short half-life of ZFP36L1 protein (<30 min) is comparable to that of a well-characterized, inducible transcription factor, c-Myc (Figure S2M), suggesting that continuous translation is required to maintain elevated protein expression. Since the transcription inhibitor actinomycin D blocked ZFP36/L1 protein induction upon FBS stimulation (Figure S2N), we postulate that the sequence of events leading to elevated ZFP36 protein is as follows: growth factors activate RTK-MAPK signaling, thereby initiating *de novo* Zfp36 mRNA transcription and translation, where the translation is regulated, at least in part, by mTORC1/2 activity (Figure S2O). Collectively, these data are consistent with ZFP36 proteins behaving as immediate-early genes.

In order to study the role of ZFP36 induction in growth factor-stimulated cells, we reasoned that ablation of each ZFP36 family member simultaneously would be necessary to observe a functional consequence given their potential redundant and compensatory roles.^17–19^ Accordingly, we established immortalized MEFs derived from mice engineered with *LoxP* sites flanking each Zfp36 family member allele. Administration of increasing adenoviral-packaged CRE recombinase (adeno-Cre) or GFP control (adeno-GFP) revealed dose-dependent Zfp36 family member excision within 24-h post-infection (Figure S3A). We optimized adeno-Cre multiplicity of infection (MOI) based on loss of Zfp36, Zfp36l1, and Zfp36l2 mRNA expression (Figures S3B and S3C) and confirmed loss of ZFP36 and ZFP36L1 protein expression in the adeno-Cre-infected, FBS-stimulated Zfp36/l1/l2 triple-floxed MEFs (Figure S3D). Together, these data provide evidence that complete elimination of all ZFP36 family member genes is readily achievable in our Zfp36/l1/l2 triple-floxed MEFs.

### eCLIP-seq reveals ZFP36 binding to metabolism-related mRNAs

**Figure 2.**
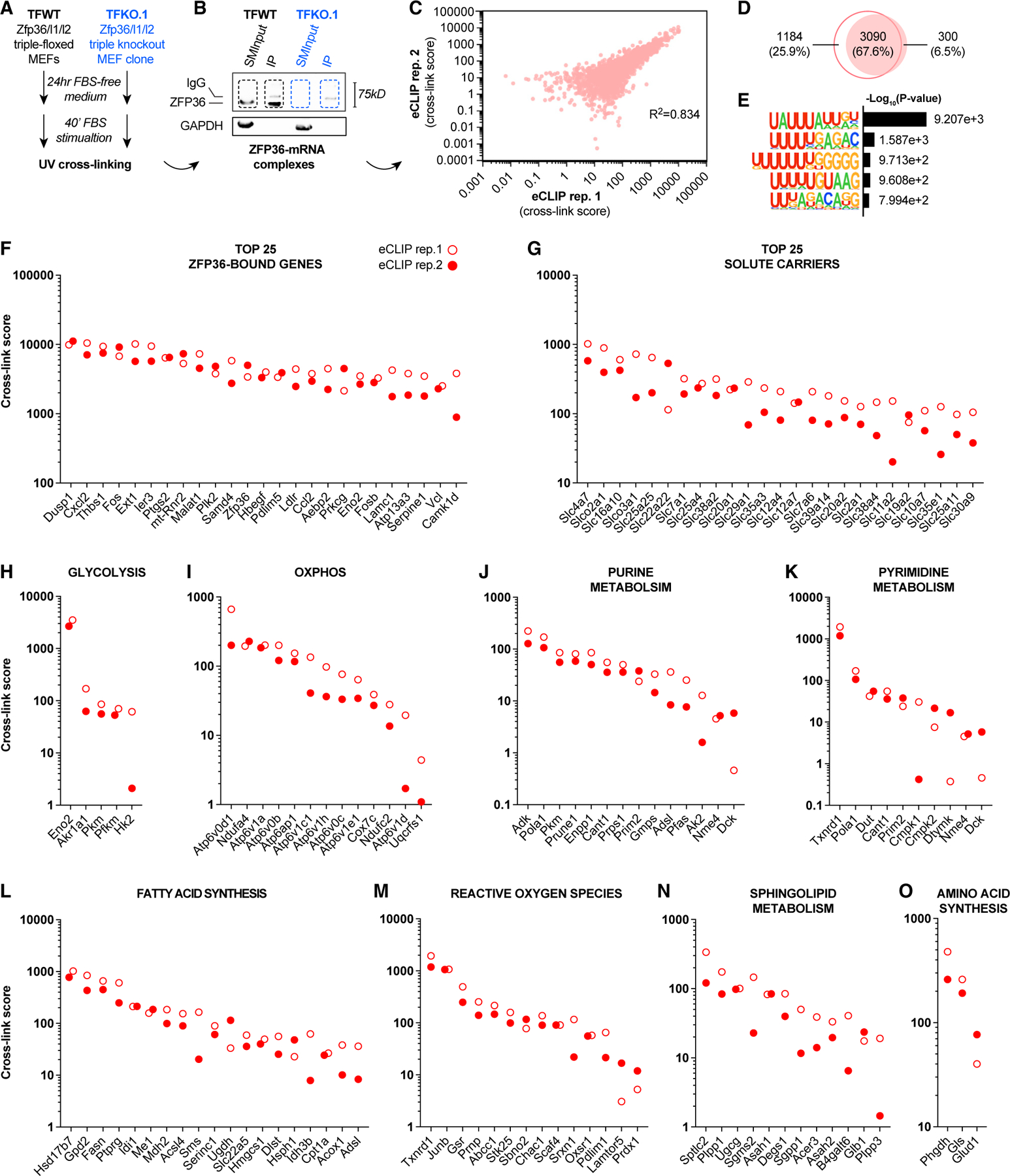
eCLIP-seq analysis reveals ZFP36 binding to metabolism-related mRNAs (A) Schematic diagramming MEF cell lines and culture conditions used for eCLIP-seq library preparation. (B) SDS-PAGE immunoblot of UV cross-linked ribonucleoprotein complexes. Input and ZFP36-immunoprecipitated fractions from ZFP36/L1/L2 wild-type (TFWT) or triple knockout MEF clone (TFKO.1) were immunoblotted for ZFP36 and GAPDH. (C) Score correlation per gene between eCLIP-seq replicate 1 and 2. Pearson correlation coefficient shown. (D) Overlapping and unique genes from each eCLIP-seq replicate. (E) HOMER Motif enrichment analysis of ZFP36 binding sites within target mRNAs identified by eCLIP-seq in both replicates. (F–O) High-confidence putative ZFP36 target mRNAs identified in both eCLIP-seq ZFP36/L1/L2 WT (TFWT) replicates, excluding peaks identified in ZFP36/L1/L2 TKO (TFKO.1) replicates, and ranked by UV cross-link score. Data curation was performed using KEGG gene sets from Molecular Signatures Database (MSigDB).

To unbiasedly identify direct ZFP36-mRNA interactions at the transcriptome-wide level during acute growth factor signaling, we performed enhanced UV cross-linking immunoprecipitation sequencing (eCLIP-seq) in biological duplicates. This molecular technique covalently preserves ribonucleoprotein complexes enabling immunoprecipitation of ZFP36 and subsequent sequencing of prepared libraries to determine precise binding site locations at single-nucleotide resolution.^20^ For these eCLIP-seq experiments, we used Zfp36/l1/l2 triple-floxed wild-type (TFWT) MEFs as well as an adeno-Cre-infected Zfp36/l1/l2 triple knockout (TKO) MEF clone (TFKO.1). TFKO.1 cells controlled for off-target affinities of the ZFP36 antibody. To carryout eCLIP-seq, TFWT and TFKO.1 MEFs were plated in regular growth conditions, serum starved overnight, and then serum stimulated for 40 min to induce ZFP36 protein expression prior to UV cross-linking (Figure 2A). Immunoblots of cellular fractions from the TFWT or TFKO.1 MEFs show comparable size-matched input (SMInput) material used for eCLIP-seq normalization, and anti-ZFP36 antibody immunoglobulin G (IgG) detection, as well as lack of ZFP36 signal in the TFKO.1-specific lanes (Figure 2B). We then excised and sequenced these ZFP36-mRNA complexes, quantitatively scoring each interaction. mRNAs identified in each replicate of the TFKO.1 immunoprecipitation (IP) libraries were minimal (≤30) and excluded from subsequent analyses (Figures S3E and S3F). Correlating the score of each eCLIP-seq duplicate on a gene-by-gene basis revealed highly reproducible (R^2^ = 0.834) results across 3,090 commonly bound mRNAs (Figures 2C and 2D). Impressively, our first eCLIP-seq replicate captured more than 90% of the genes bound by ZFP36 in the second trial. Moreover, the classical categorization of ZFP36 as an RBP that preferentially targets AREs was recapitulated in our hands as indicated by a 5.8-fold enrichment of the UAUUUAUU motif relative to competing binding sites (Figure 2E).^21,22^ Our high-confidence binding map of putative ZFP36 target mRNAs also identified known ZFP36 target genes such as Dusp1, Cxcl2, and Ptgs2, which were tiered in the top 25 cross-link scores (Figure 2F).^23–26^ Unbiased KEGG pathway enrichment analysis confirmed previously described migratory- and cytokine-related regulatory networks, with proteoglycans in cancer, focal adhesion, and tumor necrosis factor (TNF) signaling pathways representing the top three unbiased hits (Figure S3G)^26^.

Intriguingly, among known ZFP36 target genes and the cellular processes they participate in, our eCLIP-seq analysis also revealed ZFP36 binding to a large number of mRNAs encoding metabolic enzymes, nutrient transporters, and nutrient sensors (Figures 2F–2O). This was consistent with MAPK, PI3K-Akt, and mTOR KEGG pathway enrichments and further supported by Molecular Function Gene Ontology (GO) terms such as small GTPase binding, Ras GTPase binding, and protein serine/threonine kinase activity (Figures S3H and S3I). Similarly, Biological Process GO terms revealed positive regulation of cell projection organization and neuron projection development, consistent with regulation of cellular metabolism (Figure S3J). We systematically categorized the metabolism-related mRNAs bound by ZFP36 in our eCLIP-seq analysis within common pathways, including glycolysis (Figure 2H), oxidative phosphorylation (Figure 2I), purine and pyrimidine metabolism (Figures 2J and 2K), fatty acid synthesis (Figure 2L), reactive oxygen species (Figure 2M), sphingolipid metabolism (Figure 2N), and amino acid synthesis (Figure 2O). Among the ZFP36-bound mRNAs we identified were those encoding critical regulatory enzymes that likely influence overall pathway flux. For example, ZFP36 bound to mRNAs encoding key enzymes involved in fatty acid biosynthesis (Fasn), serine synthesis (Phgdh), glutaminolysis (Gls), sphingolipid biosynthesis (Sptlc2), and glycolysis (Hk2, Pfkm, Pkm).^27–32^ Moreover, Slc2a1, the primary glucose transporter in proliferating and cancer cells, was also found bound by ZFP36 (Figure 2G). Given the number of bound nutrient transporter mRNAs belonging to the SLC gene superfamily (>40), we presented the top 25 scoring, assigning the bicarbonate transporter Slc4a3 as the top hit, which was recently shown to be limiting for nucleotide synthesis (Figure 2G).^33^

**Figure 3.**
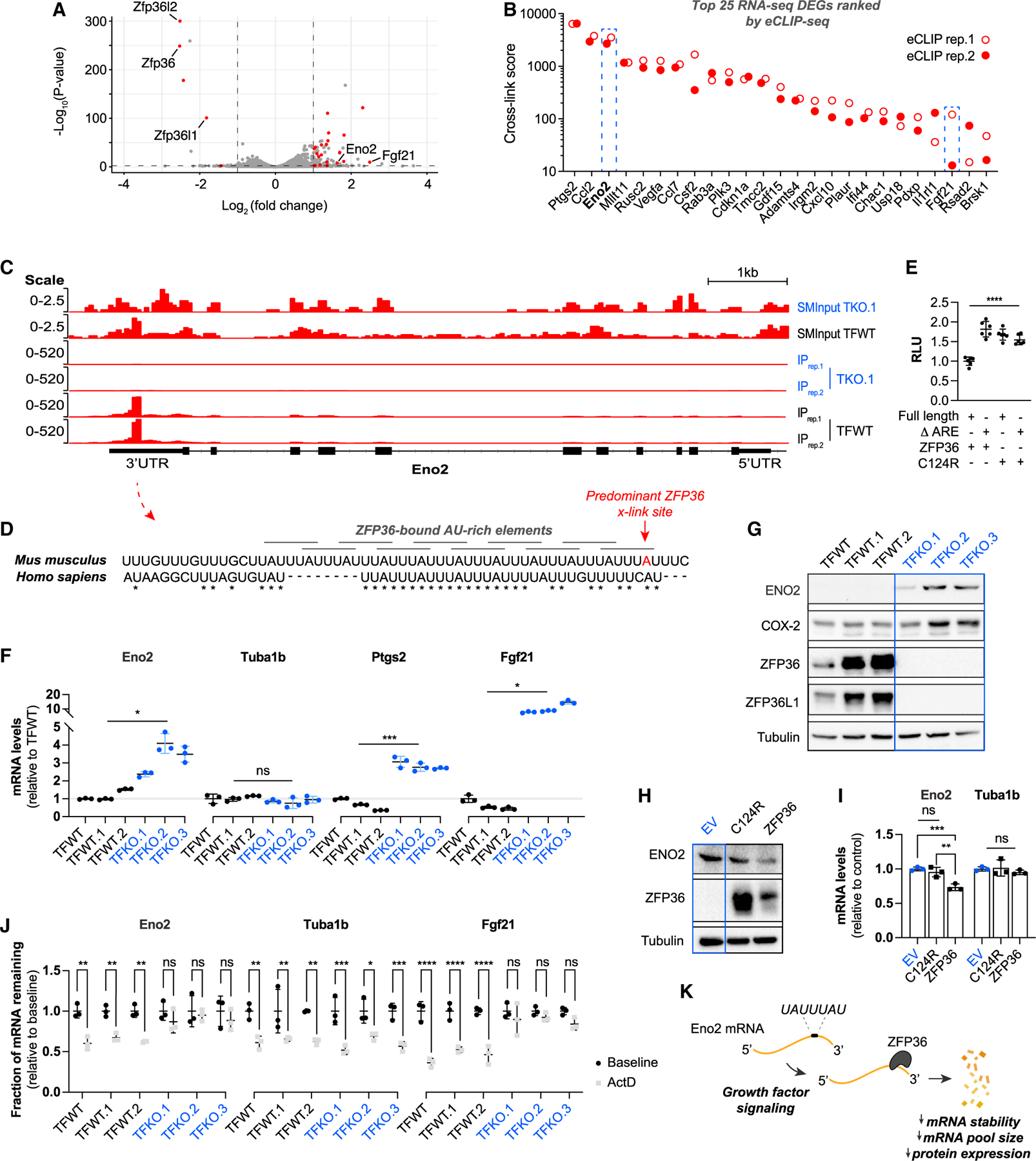
ZFP36 directly binds and promotes decay of Eno2 mRNA (A) RNA-seq volcano plot of adeno-GFP-treated (TFWT) vs. adeno-Cre-treated (TFKO) Zfp36/l1/l2 triple floxed MEFs stimulated for 1 h with 10% FBS following overnight serum deprivation. Dashed lines indicate adjusted p ≤0.01 or log_2_(fold change) ≥1 or ≤−1. Red colored dots represent significantly differentially expressed mRNAs also bound by ZFP36 in eCLIP-seq experiment. (B) Top 25 upregulated transcripts in ZFP36/L1/L2 TKO (TFKO) MEFs identified by RNA-seq from serum-starved and -stimulated culture conditions that are also bound by ZFP36. Genes are ranked by eCLIP-seq cross-link score. (C) Integrative Genomics Viewer generated from eCLIP-seq experiments showing the ZFP36 binding site on Eno2 mRNA within 3′ UTR. (D) Clustal Omega Multiple Sequence Alignment of the ZFP36 target binding sequence within human and mouse Eno2 3′ UTRs. AREs are annotated in overlapping gray bars, identities are indicated by asterisks (*), and predominant ZFP36 cross-link sites within mouse Eno2 mRNA are colored red. (E) Luciferase activity in HeLa cells cotransfected with either full-length Eno2 3′ UTR luciferase reporter or one in which the AREs have been deleted (Δ ARE), CMV-driven WT ZFP36 or ZFP36^C124R^ mutant deficient in RNA binding, and Renilla luciferase (n = 6). Data are normalized to Renilla luciferase and presented relative to full-length Eno2 3′ UTR in the presence of WT ZFP36. (F and G) Relative transcript (F) or protein (G) levels of indicated genes compared between Zfp36/l1/l2 triple-floxed WT MEFs (TFWT) and clones derived from TFWT MEFs treated with adeno-GFP (TFWT.1,2) or adeno-Cre (TFKO.1–3) and single cell expanded. (H and I) Immunoblot (H) or qRT-PCR (I) of Eno2 or Tuba1b in a ZFP36/L1/L2 TKO MEF clone (TFKO.1) transduced with CMV-driven empty vector (EV) control, human zinc finger mutant ZFP36 (C124R), or WT human ZFP36 (ZFP36). (J) Transcript stability of Eno2, Tuba1b, or Fgf21 measured by qRT-PCR in ZFP36/L1/L2 WT (TFWT) or TKO (TFKO) MEF cell clones. Cells were deprived of serum for 24 h and then stimulated with 10% FBS for 1 h before being harvested (control) or subsequently treated for an additional 4 h with actinomycin D (ActD; 5 mg/mL). Data are presented as fold change of ActD (1-h FBS + 4-h ActD) relative to control (1-h 10% FBS-stimulated conditions) for each respective clone. (K) Model of direct ZFP36-dependent regulation of Eno2 mRNA downstream of growth factor signaling. All experiments were performed with biological replicates. Error bars denote SD (n = 3, unless otherwise indicated). *p < 0.05; **p < 0.01; ***p < 0.001.

To examine whether ZFP36 binding to metabolic transcripts affects metabolic gene expression levels, we used RNA-seq in serum-deprived baseline culture conditions or 1-h post-FBS stimulation—an intentionally delayed time point relative to our eCLIP-seq conditions to allow time for ZFP36-mediated mRNA decay—and examined how levels of ZFP36-bound mRNAs change in the presence vs. absence of ZFP36. For this analysis, we profiled mRNA levels from ZFP36/L1/L2 triple-floxed MEFs acutely treated with adeno-Cre (TFKO) or -GFP (TFWT) control for 24 h (Figure S4A). As expected, all three Zfp36 family members were among the most downregulated transcripts in the TFKO cells and were also found to be bound by ZFP36 in our eCLIP-seq analysis, consistent with their autoregulatory capacity as previously reported (Figure 3A).^34^ Conversely, we observed a general overrepresentation of ZFP36-bound mRNAs among the upregulated mRNAs in TFKO cells, consistent with the mRNA decay function of ZFP36 family members (Figure 3A). Principal-component analysis assigned 5% total expression variance to ZFP36 status, while the remaining 95% was attributed to FBS-dependent changes in gene expression (Figure S4B). Importantly, this variance was evident in both serum-deprived and -stimulated conditions, suggesting that ZFP36 family member dependencies, although likely distinct in a subset of their constituents, are measurable in the absence of growth factor signaling. This hypothesis was further supported through unbiased hierarchical clustering of significantly differentially expressed genes in serum-deprived or -stimulated conditions, revealing that top FBS-induced transcripts in TFWT cells become significantly divergent from TFKO cells upon FBS stimulation; transcripts less responsive to FBS stimulation appear to exhibit divergence at baseline (Figures S4C–S4E). Further, given the representation of SLC genes within our eCLIP-seq dataset, we chose to present distinct expression clusters of this gene family, identifying significant upregulation of 4/5 or 3/5 of the highest scoring ZFP36-bound SLC mRNAs in serum-deprived or -stimulated contexts, respectively (Figures S4E–S4G). Together, these data support the conclusion that ZFP36 binds to many target transcripts, resulting in a decrease in their relative expression levels.

### ZFP36 directly binds and promotes decay of ENO2 mRNA

To more closely examine ZFP36’s regulation of target mRNAs, we aggregated all upregulated transcripts in each RNA-seq condition (TFKO vs. TFWT; serum deprived and -stimulated) that exceeded a log_2_ fold change of 1 and a p value threshold of 0.01. Cross-referencing these genes with our ZFP36 eCLIP-seq list revealed transcripts that are particularly sensitive to ZFP36-mediated mRNA decay, encompassing several known ZFP36 target genes such as Ptgs2, Csf2, and Fgf21 (Figure 3B).^16,23,35^ Importantly, these genes span cross-link scores ranging from 13 to 6,400, suggesting that expression levels of low- and high-scoring mRNAs identified in our eCLIP-seq list can be influenced by ZFP36 binding and are likely a summation of converging regulatory inputs.

In support of the hypothesis that ZFP36 regulates metabolic enzymes, we identified the glycolytic enzyme Eno2 as among the highest eCLIP-seq-ranked differentially expressed transcripts in our integrated eCLIP- and RNA-seq datasets (Figure 3B). To verify a direct relationship between ZFP36 and Eno2, we first validated ZFP36 interaction with Eno2 mRNA using CLIP-qPCR (Figure S4H). Next, we examined the eCLIP-seq-derived binding site of ZFP36 within the Eno2 transcript using Integrative Genomics Viewer software. We found that ZFP36 bound Eno2 at a precise location within its 3′ UTR characterized by ten overlapping AREs largely conserved in mouse and human (Figures 3C and 3D). No binding was observed in the TFKO.1 ZFP36/L1/L2 KO cells, confirming that signal resulted from ZFP36 binding to the Eno2 3′ UTR, not from off-target affinities of the ZFP36 antibody used for eCLIP-seq. Next, to examine whether ZFP36 binding to the AREs in the Eno2 3′ UTR impacts Eno2 gene expression, we generated reporter constructs with luciferase cDNA fused to either the full-length Eno2 3′ UTR sequence or one lacking the eCLIP-seq-identified ARE-binding sites and measured luminescence in the presence of exogenous WT ZFP36 or a zinc finger mutant ZFP36 (ZFP36^C124R^) deficient in RNA binding. Luciferase activity was significantly elevated when the Eno2 3′ UTR reporter construct lacked AREs or when the zinc finger mutant ZFP36 was coexpressed (Figure 3E). These data suggest that direct ZFP36 binding to the AREs within the 3′ UTR of Eno2 transcripts regulates ENO2 expression levels.

To examine whether ZFP36 impacts Eno2 expression via direct mRNA decay, we single cell-expanded multiple ZFP36/L1/L2 WT (TFWT) or KO (TFKO) clonal MEF cell lines to measure relative Eno2 expression levels and stability. We observed a 2- to 4-fold increase in Eno2 mRNA in the absence of ZFP36 family members, which is similar in magnitude to expression of known ZFP36 target genes, Ptgs2 and Fgf21 (Figure 3F). Notably, Tuba1b, a gene not bound by ZFP36 within our eCLIP-seq dataset or differentially expressed in the RNA-seq, effectively served as a negative control (Figure 3F). We found that the increased Eno2 transcript levels in the TFKO MEF clones corresponded to increased ENO2 protein expression (Figure 3G). Importantly, expression of ZFP36, but not ZFP36^C124R^, was sufficient to reduce ENO2 expression in the TFKO cells (Figures 3H and 3I).^36^ These data suggest direct regulation of Eno2 mRNA expression by ZFP36 RNA binding. To examine whether ZFP36 regulates Eno2 mRNA expression via altering Eno2 mRNA stability, we treated TFWT or TFKO MEF clones with actinomycin D, a pan-transcriptional inhibitor, and compared mRNA levels of Eno2 with that of a known ZFP36 target gene, Fgf21, and a negative control, Tuba1b. We reasoned that in the context of transcription inhibition, changes in mRNA levels would likely be due to changes in mRNA stability. As shown in Figure 3J, Eno2 mRNA stability exhibited a striking dependence on the ZFP36 family, comparable to that of Fgf21. However, Tuba1b mRNA levels decreased similarly across all clones tested, providing evidence that mRNA decay was occurring in all conditions and was not dependent on the ZFP36 family (Figure 3J). Together, these results suggest that ZFP36 directly regulates ENO2 expression via promoting Eno2 mRNA decay (Figure 3K).

### ZFP36 proteins regulate cellular metabolism

**Figure 4.**
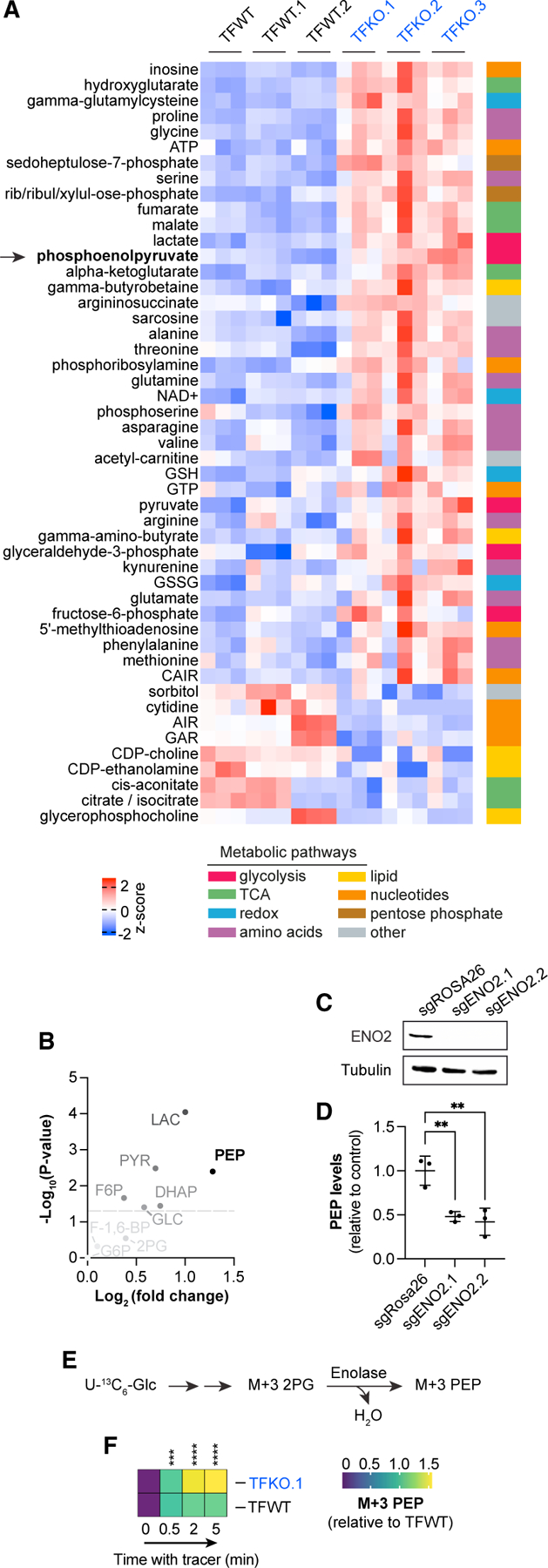
ZFP36 proteins regulate cellular metabolism (A) Targeted LC-MS metabolomics measurements comparing ZFP36/L1/L2 WT vs. TKO clones. Data are grouped by genotype, prefiltered (log2 fold change [L2FC] ≥ 0.2 or ≤ −0.2; p < 0.01), and ranked by statistically significant differential metabolite abundance, then visualized as *Z* scores across rows. (B) Volcano plot comparing glycolytic metabolite levels in ZFP36/L1/L2 triple-floxed KO MEFs (TFKO.1) vs. Zfp36/l1/l2 triple-floxed WT (TFWT) MEFs. (C) Schematic diagramming uniformly labeled [13C] glucose tracing through the enolase enzyme and the relevant isotopologues. (D) LC-MS measurements of relative levels of U-13C_6_-glucose-derived M+3 phosphoenolpyruvate (PEP) over time (0, 0.5, 2, or 5 min) in Zfp36/l1/l2 triple-floxed WT MEFs (TFWT) vs. ZFP36/L1/L2 triple-floxed KO MEFs (TFKO.1) cultured in 10% FBS growth medium. Data are normalized to TFWT cells at 0.5-min time point. All experiments were performed with biological replicates. Error bars denote SD (n = 3). *p < 0.05; **p < 0.01; ***p < 0.001.

Given the abundant metabolic mRNAs bound by ZFP36 (Figure 2) and validation that these interactions can lead to changes in expression levels (Figure 3), we next wondered whether ZFP36 impacts cellular metabolism downstream of growth signals. To examine potential regulation of metabolism by ZFP36 family members, we used liquid chromatography-mass spectrometry (LC-MS)-based metabolomics to measure pool sizes of metabolites extracted from FBS-stimulated ZFP36/L1/L2 TFWT or TFKO MEF clones. Indeed, we observed extensive ZFP36/L1/L2-dependent changes in the cellular metabolome, spanning diverse pathways within central carbon metabolism (Figure 4A). Intriguingly, levels of phosphoenolpyruvate (PEP), the forward reaction product of ENO2, are elevated in TFKO cells, consistent with the observed elevation in ENO2 protein expression (Figures 4A and 3G). Metabolites from many different pathways were elevated in the context of ZFP36/L1/L2 KO, consistent with the observed binding of ZFP36 to various nutrient transporters and mRNAs from diverse metabolic pathways. Conversely, the metabolites that exhibited decreased levels in the context of ZFP36/L1/L2 KO were mainly from specific metabolic pathways involving lipid, nucleotide, and TCA cycle metabolism. We observed downregulation of choline- and ethanolamine-related metabolites as well as specific nucleotide (AIR and GAR) or nucleoside (cytidine) species. Together, these data suggest that ZFP36 proteins broadly regulate cellular metabolism in response to acute growth factor signaling.

Next, we examined metabolites altered in ZFP36/L1/L2 KO MEF clones at the pathway level, comparing all detectable glycolytic intermediates, to gain insight into the specificity of elevated PEP levels. We found that PEP was the most elevated metabolite within glycolysis, with more than a 1.25 log_2_ fold change increase (Figure 4B). CRISPR-Cas9-mediated ablation of ENO2 in TFKO.1 cells reversed PEP accumulation (Figures 4C and 4D). Furthermore, kinetic U-13C-glucose tracing in ZFP36/L1/L2 TFWT vs. TFKO.1 MEFs revealed faster accumulation of M+3 PEP in the TFKO.1 cells (Figures 4E and 4F). These data suggest that ZFP36 family member loss increases cellular activity of enolase, consistent with the observed increase in ENO2 expression in these cells (Figure 3G).

### ZFP36 regulation of ENO2 expression occurs during retinal angiogenesis in murine neonates

**Figure 5.**
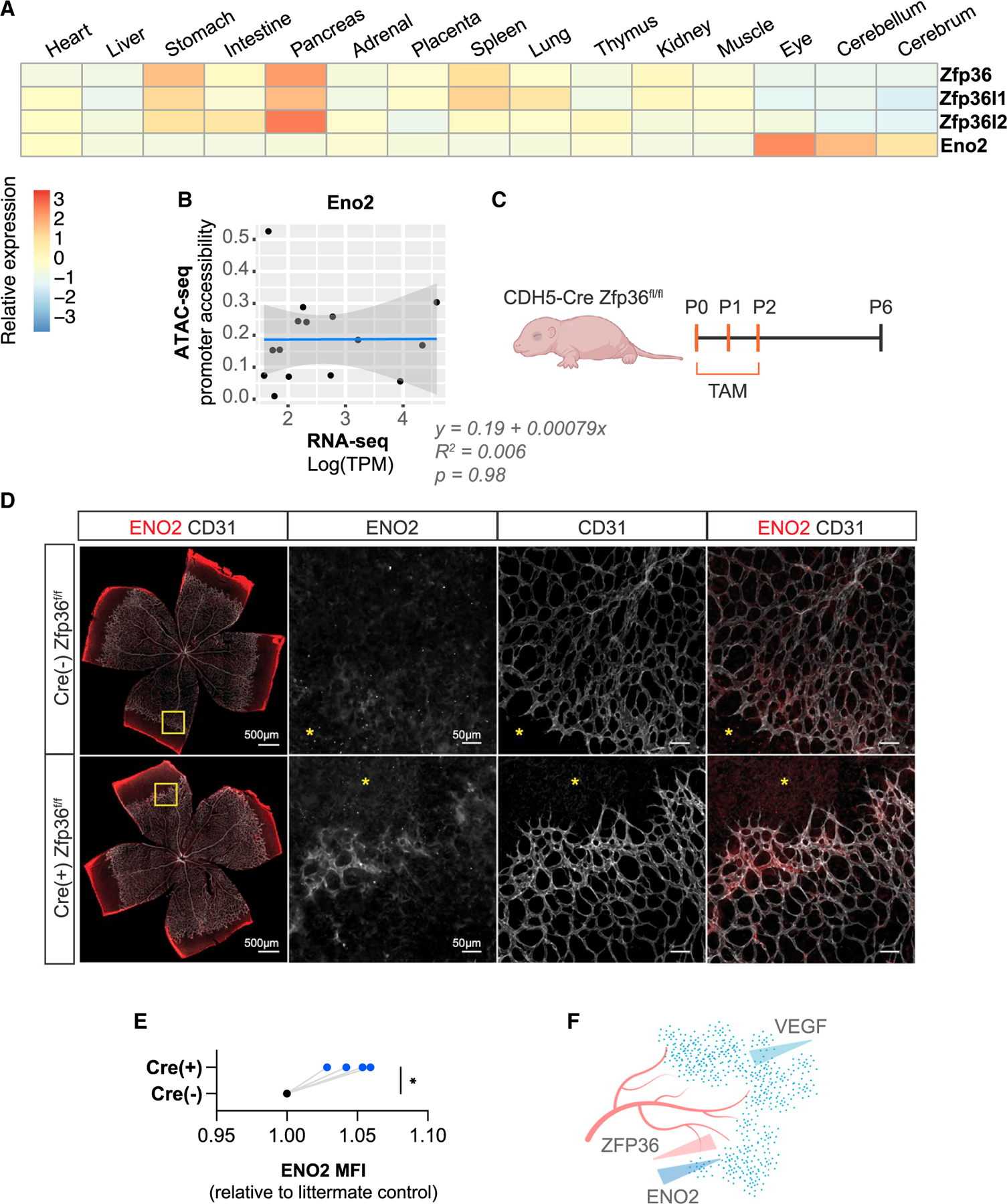
ZFP36 regulation of ENO2 expression occurs during retinal angiogenesis in murine neonates (A) Relative Eno2 expression levels from human fetal single-cell RNA-seq atlas across 15 different tissues. (B) Lack of correlation between Eno2 expression in single cell RNA-seq and Eno2 ATAC-seq data across tissues in human fetuses ranging from 89 to 125 days post-conception. (C) Schematic diagramming tamoxifen (TAM)-inducible endothelial-specific Zfp36 KO in CDH5-Cre Zfp36^fl/fl^ neonatal mice. (D) Retina immunostaining of Cre(−) or Cre(+) neonates corresponding to endothelial cell-specific ZFP36 WT or KO respectively. Retinas were stained for endothelial-specific CD31 marker or ENO2. (E) Quantification of ENO2 signal comparing Cre(−) and Cre(+) neonatal paired littermates. (F) Model of ZFP36 regulation of ENO2 expression downstream of VEGF signaling during retinal angiogenesis in murine neonates.

To examine whether ZFP36 regulates Eno2 mRNA expression in response to growth factor signaling in a physiological setting *in vivo*, we evaluated ENO2 levels in a developmental model of retinal angiogenesis where Zfp36 was deleted in the endothelial compartment.^37^ We chose to examine potential ENO2 regulation by ZFP36 in endothelial cells *in vivo* since our eCLIP-seq GO term Biological Processes analysis identified various aspects of cell projections and differentiation driven by ZFP36 regulation, which is characteristic of blood vessel-neuron crosstalk during development (Figure S3J).^38,39^ We also identified enrichment of Eno2 in the eye and general discordance between its promoter accessibility (±1 Kb transcription start site [TSS]) and mRNA expression levels in integrated human fetal single-cell assay for transposase-accessible chromatin (ATAC)- and RNA-seq datasets (Figures 5A and 5B).^18,40^ Therefore, we reasoned that developmental processes in retinal tissue specifically may rely on post-transcriptional mechanisms pacing a switch between metabolic activation states. We first confirmed ZFP36 KO by TdTomato reporter expression, which is a proxy for endothelial cell-specific Zfp36 allele recombination (Figure S5A). Indeed, we found increased ENO2 expression in ZFP36 KO endothelial cells localized specifically at the retina angiogenic front—a region previously described to be rich in VEGF—indicating that ZFP36 regulates ENO2 expression in the angiogenic front *in vivo* (Figures 5C–5F).^41^ Consistently, we found that cultured HUVECs induce Zfp36 expression in response to VEGF stimulation (Figure S1A). Taken together, these data are consistent with growth factor-dependent induction of ZFP36 in endothelial cells in the developing vasculature impacting metabolic gene expression within retinal tissue.

## DISCUSSION

### ZFP36-mediated mRNA decay as a mechanism of rapid metabolic response

In this study, we demonstrate rapid upregulation of the ZFP36 family of RNA-binding proteins upon acute growth factor signaling. We provide evidence that ZFP36 directly binds to metabolic enzyme and transporter mRNAs and show that ZFP36 interaction with one bone fide mRNA target, Eno2, results in reduced ENO2 expression through a direct mRNA decay mechanism. Finally, we provide evidence that Eno2 is regulated by ZFP36 *in vivo* in a developmental model of VEGF-stimulated retinal angiogenesis. We therefore propose ZFP36-mediated mRNA stability as a critical mode of rapid metabolic regulation downstream of growth factor signaling.

The ZFP36 family of decay factors are fitting candidates to mediate rapid cell-autonomous metabolic reprogramming given their onset of expression within minutes following a growth factor stimulation. Previous evidence recognizes the requirement for ZFP36-mediated regulation of genes within acute and resolving inflammatory contexts, which are known to be facilitated by underlying metabolic transitions. For example, TNF-a is the gold standard gene shown to be regulated by ZFP36 in LPS-activated macrophages.^42^ Later studies expanded the direct ZFP36 binding repertoire to include dozens of inflammatory cytokines across diverse cell lineages. Also found within these datasets are metabolic enzymes whose activities similarly span diverse pathways. Indeed, whole-body KO mouse models for ZFP36 lead to cachexia, while ablation of all three ZFP36 family members is embryonic lethal—a characteristic of metabolic dysregulation. Further, our study directly links ZFP36 to Eno2 mRNA stability.^43,44^ We posit that ZFP36-mediated mRNA decay acts on a temporal and stoichiometric basis, targeting genes not only with differential affinity—perhaps due to their mere number of AREs—but also within a combinatorial system dictated by relative mRNA expression levels, subcellular localization, existence of secondary mRNA structures, or prior occupancy of these mRNAs by competing RBPs. Metabolic regulation is likely an underappreciated, yet key, attribute of ZFP36 function—akin to the upstream signaling pathways that induce ZFP36 expression.

The characterization of metabolites themselves participating in signaling processes has gained more appreciation in recent years.^45^ Relevant to the findings presented here, PEP insufficiency culminates in defects in Ca^2+^-NFAT signaling and cytokine secretory profiles of T cells affecting their anti-tumor effector function.^46^ Furthermore, PEP has been postulated as a high-energy phosphate donor, which could account for some of its biological function more broadly through mechanisms analogous to ATP.^47^ In a separate but potentially related study, prostaglandin (PG) lipid mediators that rely on COX-2 for their synthesis are impinged upon by the cell-permeable itaconate derivative 4-octyl itaconate (4-OI) to limit their production. 4-OI was shown to decrease Ptgs2/COX-2 mRNA and protein levels independent of NRF2 activity.^48^ It is intriguing to speculate that ZFP36 may impact these mechanisms through direct targeting of Eno2 or Ptgs2, as their protein product enzymatic activities produce PEP and PGs, respectively.

While our study confirmed ZFP36 regulation of Eno2 mRNA stability and ENO2 expression (Figure 3), we found that ZFP36 directly binds to dozens of transcripts encoding nutrient transporters and metabolic enzymes (Figure 2). Consistently, we found that ZFP36/L1/L2 TKO impacts levels of metabolites from diverse metabolic pathways (Figure 4). Together, these data suggest that ZFP36 may simultaneously impact many metabolic pathways in response to growth factor signaling and may serve to broadly coordinate the cellular metabolic response to external stimuli.^49^

### ZFP36 proteins participate in a growth-suppressive negative feedback module

ZFP36 proteins have been postulated to act as tumor suppressors.^50,51^ A large proportion of ARE-containing transcripts are considered mitogenic, and ZFP36 loss leads to a rise in their abundance.^52^ Interestingly, although we observed ZFP36 family member induction in response to growth factor stimuli within cancer cell lines, the dynamic range of expression was modest relative to normal immortalized cells of similar origin. This finding is consistent with the observation that aged mice decrease ZFP36 compared with their younger counterparts.^53^ Moreover, the well-known protooncogene c-Myc transcriptionally represses ZFP36 and is selected for in various malignancies including, but not limited to, B cell lymphomas.^54^ We raise the possibility of ZFP36 as a pharmacologic target for stabilization to exploit its pleiotropic affects and inherent biological specificity in various cell growth processes.

Physiological activities during organismal development depend on growth pathways that are also used by cancer cells. Cells must engage in these pathways to contract migratory or proliferative behavior to achieve homeostasis. Regulation occurs with tissue specificity, and the extent to which ZFP36 proteins participate has not been fully elucidated.^7,55^ We observe one such biological context—a neonatal murine model of retinal angiogenesis—that exhibits post-transcriptional suppression of ENO2 levels by ZFP36. Previous studies suggest that endothelial cells engage glycolysis during angiogenesis.^56^ It is not clear the extent to which ZFP36 ablation would promote or inhibit angiogenesis, as our observations were limited to identifying the impact of ZFP36 loss on Eno2 gene expression. It is conceivable, however, that endothelial tissue preferentially engages ZFP36-mediated decay to constrain uncontrolled growth. The direct binding of ZFP36 to specific metabolism-related mRNAs, including Eno2, warrants further investigation to uncover the extent to which these interactions result in phenotypic consequences.

### ZFP36 proteins are constituents of a broader biological paradigm

This mechanistic link between mRNA decay and rapid metabolic responses—more closely examined for the glycolytic enzyme ENO2—provides evidence that cells and tissues reserve multiple levels of regulation to accomplish biological outcomes. The rapid onset of ZFP36 family member-mediated decay in response to growth signals adds an additional facet to a long-standing dogma of transcriptional or post-translational regulatory modules. However, these modes of regulation are clearly not disparate in their action as evident by their role in mediating ZFP36 expression onset through upstream kinases, as well as the numerous phosphorylation sites within ZFP36 proteins themselves that influence their mRNA-binding capacity or protein-protein interactions.^57^ Since ZFP36 proteins require cooperation with deadenylase complex members to carry out mRNA decay, post-translational modifications on ZFP36 family members are likely central to the broader biological process at play. Our data suggests that cellular metabolism, in addition to being regulated at the transcriptional and post-translational level, is also regulated via the post-transcriptional toggling of mRNA levels and that rapid cellular metabolic responses to external stimuli are a product of coordinated multi-dimensional regulatory processes.

### Limitations of the study

Many genes are induced by growth factor signaling. Our focus on ZFP36 family proteins and their role in metabolic regulation given our eCLIP-seq findings is an intentional bias of this study since other growth factor-induced genes likely also play a role. Our study mechanistically dissects ZFP36 binding to metabolic genes and regulation of one metabolic target gene, Eno2, as well as the consequence of this regulation primarily in culture conditions amendable to experimental manipulation. We acknowledge that *in vivo* cellular metabolism is dependent on the surrounding tissue environment and that *in vitro* cell culture does not recapitulate physiological scenarios. We assert that highly conserved gene regulatory circuits are less dependent on such artifacts, though the extent to which phenotypic outcomes are impacted is less clear. It is this perspective that authenticates the direct ZFP36-mRNA binding interactions revealed by our investigation, warranting further investigation as to the relative weight of these relationships to dictate cellular responses on a case-by-case basis. Although we provide evidence that the ZFP36 target gene ENO2 was a predictable relationship generated by our studies, it may not be a universal outcome of ZFP36 loss.

Experimental tools and model systems are often limiting for dissecting biological processes. To this end, we reasoned that ablating all three ZFP36 family members simultaneously would avert redundancy among gene family paralogs and the potential for concluding false negatives in regard to target gene regulation. Indeed, DepMap Portal analyses identify distinct and overlapping codependencies for each ZFP36 family member, but the extent to which genetic manipulation of a single family member impacting another has not been fully explored. Further, by employing ZFP36/L1/L2 TKO control cells to generate background eCLIP-seq signal, the selective rise ZFP36/L1/L2 target genes may also raise the threshold for identifying true targets. A similar argument can be made for the general over-representation of metabolic enzyme mRNAs within cells. Although eCLIP-seq addresses this technical artifact by normalizing to a SMInput sample—which we included for each genotype and replicate performed—we nonetheless allow for the possibility that background transcriptome levels influence the signal-to-noise ratio for the eCLIP-seq analytical methodology used in this study.

## STAR★METHODS

### RESOURCE AVAILABILITY

#### Lead contact

Further information and requests for resources and reagents should be directed to and will be fulfilled by the lead contact, Heather R. Christofk (hchristofk@mednet.ucla.edu).

#### Materials availability

Materials used in this study are commercially available.

#### Data and code availability

RNA-seq (SRA: PRJNA942602) and eCLIP-seq (SRA: PRJNA943291) data have been deposited at NCBI Sequence Read Archive (SRA) and are publicly available as of the date of publication. Accession numbers are listed in the key resources table. This paper does not report original code. Any additional information required to reanalyze the data reported in this paper is available from the lead contact upon request.

### EXPERIMENTAL MODEL AND SUBJECT DETAILS

#### Mouse experiments

##### Insulin stimulation

Wildtype C57BL/6 mice were housed in pathogen-free animal facilities at UCLA in accordance with the UCLA Institutional Animal Care and Use Committee (IACUC). All animal experiments were approved by the UCLA Animal Research Committee (ARC), and were compliant with all relevant ethical regulations. Mice were maintained on a 12-h light/12-h dark cycle. On the day of the experiment, mice were fasted for 5-h, then insulin was dissolved in water and administered (2U/kg) via intraperitoneal (IP) injection; control mice received a water vehicle IP injection only.

##### Neonatal retina angiogenesis

All animal procedures were approved and performed in accordance with Northwestern University IACUC. All mouse strains were maintained on a C57BL/6J background, both female and male mice were used for retina analysis. For inducible endothelial-specific deletion of ZFP36, Tg(Cdh5-cre/ERT2) mice^58^ were crossed with Zfp36flox/flox line.^59^ This line was further crossed with R26RTd Cre reporter lines (Gt(ROSA)26Sor tm14(CAG tdTomato)Hze).^60^ Internal littermate controls were achieved by crossing Cre(−) Zfp36flox/flox with Cre(+) Zfp36flox/flox parents. Three consecutive days of tamoxifen was administered by oral gavage (5uL, 20 mg/mL) starting on day of birth.

### METHOD DETAILS

#### MEF cell line generation

##### MEF isolation

To prepare mouse embryonic fibroblasts, embryos were obtained through time mating of Zfp36/l1/l2 triple-floxed males and females.^51,61^ Starting at E0.5, pregnant dams were followed with daily weighing until embryonic day E11.5. The dams were euthanized by cervical dislocation following isoflurane induction. Embryos were removed using sterile dissection practices and rinsed in cold PBS. The fetal heads, spinal columns, and organs were removed and the remaining tissue was processed for cell culture. The tissue was dissociated using a razor blade and TrypLE Express (Fisher) and cells were diluted in DMEM containing 15% FBS, 10 mM nonessential amino acids, 4 mM L-glutamate, 1 mM sodium pyruvate, and 1% penicillin/streptomycin. Cells from each embryo were pelleted at 300xg for 5 min, resuspended in complete medium, and plated in 10 cm tissue culture dishes. Once cells reached ~80% confluency (24–36hr), each MEF cell line was split 1:5. Several plates were cryopreserved, and the remaining cells were used for immortalization.

##### MEF immortalization

Zfp36/l1/l2 triple-floxed MEFs were seeded into 6-well dishes at ~75% confluency. The following day, MEFs were exposed to overnight SV40 transfection using Lipofectamine 3000 Reagent (Fisher Cat# L3000001), then medium was replaced with fresh complete medium for an additional 24-h. Next, MEFs were serial passaged with varying dilutions ranging between 1:3 and 1:10 to ensure survival while applying selective immortalization pressure. Once MEFs were visibly immortal – assessed by nearly 100% adherent cultures without excessive floating/dead cells (~5–10 passages) – they were cryopreserved in 20% FBS-supplemented complete medium with 5% DMSO.

##### Adenoviral-mediated MEF clonal cell line generation

Zfp36/l1/l2 triple-floxed MEFs were seeded into 6-well plates at ~60% confluency then exposed to adeno-CRE or adeno-GFP control (Log_10_(MOI) = 3.4) for 24 hours in complete medium (DMEM 10%FBS). The following day medium was replaced with fresh complete medium for 24 hours then cells were trypsinized and plated into 96-well plates at a density of ~1 cell/well. MEFs were allowed to proliferate for ~7–10 days and monitored for single cell colony outgrowth. Single cell clones were then expanded and screened for ZFP36/L1/L2 gene expression at the RNA and protein level with RT-qPCR and immunoblotting respectively. ZFP36/L1/L2 was confirmed to be absent in adeno-Cre-treated MEFs, while adeno-GFP-treated MEF clones maintained expression of ZFP36/L1/L2 proteins.

#### CRISPR-Cas9-mediated ENO2 knockout in ZFP36/L1/L2 triple knockout MEFs

Guide oligos were cloned into lentiCRISPR V2-Blast (Addgene 83480) and used to generate lentivirus.

sgROSA26: GTAGTAATGAGAGTTCACTG

sgENO2.1: ATAGAGATCCACCTCCACGG

sgENO2.2: GATTTGGGCCCGAGAGATCT

#### Cell culture conditions

All cell lines, with the exception of MCF-10A cells and HUVECs, were cultured in DMEM containing 1 mM pyruvate and 4 mM glutamine supplemented with 10% fetal bovine serum (FBS) and 1% penicillin/streptomycin in a humidified incubator at 37°C with 5% CO_2_ and atmospheric oxygen; MCF-10A cells were cultured in DMEM/F12 supplemented with 5% horse serum, 10μg/mL insulin, 10μg/mL cholera toxin, 500ng/mL hydrocortisone, and 20ng/mL epidermal growth factor (EGF); HUVECs were cultured in MCDB-131-WOFBS (VEC Technologies) with 10% FBS.

#### Cell culture treatments

##### Growth factor stimulation

Stock growth factor solutions were prepared in water in single-use aliquots and applied directly to overnight FBS-deprived cell cultures at approximately 75–85% cell confluency. In the case of 10% FBS treatments, FBS used in standard culture conditions was re-introduced. For experiments involving MCF-10A cells, all culture medium additives were omitted overnight prior to stimulations; 5% horse serum was substituted for 10% FBS.

##### Inhibitor treatments

SB2035800 (p38 inhibitor, Selleck Chemical), PD0325901 (MEK1/2 inhibitor, Sigma), Torin (mTOR inhibitor, Tocris Bioscience), cycloheximide (translation inhibitor, Sigma), and actinomycin D (transcription inhibitor, Sigma) were re-suspended in DMSO.

##### Adenoviral-GFP/CRE infection

Adenoviral-packaged GFP or CRE (Vector Biolabs) was prepared in DMEM, 2% BSA, and 2.5% glycerol storage buffer at 1×10^10^ PFU/mL. Infections were performed by directly spiking adenovirus into culture medium using single-use aliquots.

##### Lentivirus production and transduction

Lentiviral particles were produced in 293T cells by co-transfecting with a construct of interest and third-generation packaging plasmids containing *vsvg*, *gag/pol*, and *rev* sequences. Following a 48-hour incubation, lentivirus-rich medium was filtered through a 0.45μM porous membrane and applied to target cells overnight in the presence of 4 μg/mL polybrene. Next, infected cells were cultured in regular growth medium for 24-hours prior to beginning blasticidin (10μg/mL) antibiotic selection.

#### Cell lysis and immunoblotting

Cells were lysed in RIPA buffer supplemented with 2μg/mL aprotinin, 2μg/mL leupeptin, 0.7μg/mL pepstatin, 20mM sodium fluoride, 1mM sodium orthovanadate, 1mM dithiothreitol, 10mM beta-glycerophosphate, and 10mM sodium pyrophosphate. Following protein quantification using Bradford reagent, western blots were performed using standard procedures. The following commercially available antibodies were used for immunoblotting: tristetraprolin (Cell Signaling Technology 71632, 1:500), BRF1/2 (Cell Signaling Technology 2119, 1:1000), ERK (Cell Signaling Technology 9102, 1:1000), phospho-Thr202/Tyr204 ERK (Cell Signaling Technology 4370, 1:1000), S6 kinase (Cell Signaling Technology 2708, 1:1000), phospho-T389 S6 kinase (Cell Signaling Technology 9234, 1:500), c-Myc (Cell Signaling Technology 18583, 1:1000), GAPDH (Cell Signaling Technology 5174, 1:1000), β-actin (Cell Signaling Technology 3700, 1:1000) and α-tubulin (Sigma T6074, 1:10,000).

#### Site-directed mutagenesis

Site-specific mutagenesis primers for generating CMV-driven human ZFP36^C124R^ or the Eno2 3’UTR ARE deletion (Δ ARE) were designed using Agilent QuickChange Primer Design tool. High fidelity PfuTurbo DNA polymerase was used for PCR amplification per manufacturers instructions, followed by 1-hour DpnI digestion of template DNA, and propagation of mutant plasmid in One Shot Stbl3 Chemically Competent E. coli. See Table 2 for primer sequences used.

#### Quantitative real-time PCR

Total RNA was isolated from cells using the Qiagen RNeasy Kit followed by on-column DNase digestion and cDNA synthesis using iScript Supermix Kit (Bio-Rad) with 700ng RNA per reaction. Next, cDNA was diluted 5-fold and 2μl was combined with 0.5μM primers in 20μl Power SYBR master mix (Applied Biosystems) and amplified on a QuantStudio5 (Applied Biosystems). Relative transcript levels were calculated using the ΔΔCt method with Rplpo as the reference gene. Table 1 provides primer sequences used.

#### Intracellular metabolite extraction and mass spectrometry-based metabolomics

Cells were seeded into six-well plates at approximately 75% confluency. For glucose tracing experiments, 10mM U-^13^C-glucose (Cambridge Isotopes) and 10% dialyzed FBS was substituted for 25 mM D-glucose and 10% FBS respectively. At time of harvest, cells were placed on ice and washed with 150 mM ammonium acetate, pH 7.3, then 500 μL 80% methanol was added to each well and transferred to −80°C for 15 minutes. Cells were then scraped into Eppendorf tubes, vortexed vigorously and centrifuged max speed for 10 minutes at 4°C. 250 μL of supernatant was transferred to a new tube and evaporated under vacuum. Dried metabolites were then stored at −80°C until further processing detailed below.

Dried metabolites were reconstituted in 100 μL of a 50% acetonitrile (ACN) 50% dH20 solution. Samples were vortexed and spun down for 10 min at 17,000g. 70 μL of the supernatant was then transferred to HPLC glass vials. 10 μL of these metabolite solutions were injected per analysis. Samples were run on a Vanquish (Thermo Scientific) UHPLC system with mobile phase A (20mM ammonium carbonate, pH 9.7) and mobile phase B (100% ACN) at a flow rate of 150 μL/min on a SeQuant ZIC-pHILIC Polymeric column (2.1 3 150 mm 5 mm, EMD Millipore) at 35°C. Separation was achieved with a linear gradient from 20% A to 80% A in 20 min followed by a linear gradient from 80% A to 20% A from 20 min to 20.5 min. 20% A was then held from 20.5 min to 28 min. The UHPLC was coupled to a Q-Exactive (Thermo Scientific) mass analyzer running in polarity switching mode with spray-voltage=3.2kV, sheathgas=40, aux-gas=15, sweep-gas=1, aux-gas-temp=350°C, and capillary-temp=275°C. For both polarities mass scan settings were kept at full-scan-range = (70–1000), ms1-resolution=70,000, max-injection-time=250ms, and AGC-target=1E6. MS2 data was also collected from the top three most abundant singly-charged ions in each scan with normalized-collision-energy=35. Each of the resulting ‘‘.RAW’’ files was then centroided and converted into two ‘‘.mzXML’’ files (one for positive scans and one for negative scans) using msconvert from ProteoWizard. These ‘‘.mzXML’’ files were imported into the MZmine 2 software package. Ion chromatograms were generated from MS1 spectra via the built-in Automated Data Analysis Pipeline (ADAP) chromatogram module and peaks were detected via the ADAP wavelets algorithm. Peaks were aligned across all samples via the Random sample consensus aligner module, gap-filled, and assigned identities using an exact mass MS1(+/−15ppm) and retention time RT (+/−0.5min) search of our in-house MS1-RT database. Peak boundaries and identifications were then further refined by manual curation. Peaks were quantified by area under the curve integration and exported as CSV files. If stable isotope tracing was used in the experiment, the peak areas were additionally processed via the R package AccuCor 2 to correct for natural isotope abundance. Peak areas for each sample were normalized by the measured area of the internal standard trifluoromethanesulfonate (present in the extraction buffer) and by the number of cells present in the extracted well.

#### ZFP36 eCLIP-sequencing

##### Cell culture conditions and UV cross-linking

Zfp36/l1/l2 triple-floxed MEFs (TFWT) or a Zfp36/l1/l2 triple knockout clone (TFKO.1) were seeded subconfluently into 15cm dishes followed by 24-hours serum deprivation. Next, cells were stimulated for 40-minutes with FBS (10% total volume), placed on ice, and washed twice with cold PBS. After aspirating the final PBS wash, 18mL ice cold PBS was added to the culture vessel and cells were UV-irradiated once at 300mJ/cm^2^. Cells were scraped into 10mL ice cold PBS, pelleted and snap frozen in liquid nitrogen and stored at −80°C until use. An eCLIP library replicate was constructed from 12 cross-linked 15-cm plates pooled together and allocated to SMInput or IP; we constructed 8 libraries total, consisting of a SMInput and IP for each cell line in biological duplicate.

##### Antibody-bead cross-linking preparation

For each immunoprecipitation, 25μg anti-tristetraprolin antibody (Millipore ABE285) was conjugated to 125ul Protein A Dynabeads (Thermo Fisher) in binding buffer (1mg/mL in PBS) for 1hr at 4°C. Unbound antibody was removed from the beads by two washes with binding buffer. To covalently cross-link antibody to beads, dimethyl pimelimidate (DMP) was dissolved in Wash buffer (0.2 M triethanolamine in PBS) at 13mg/mL immediately prior to use. After a 5-minute wash at 4°C with Wash buffer, DMP was diluted to 0.5X with water and added to the antibody-beads mix at 1:1 ratio for 30-minutes rotating at room temperature. DMP cross-linking was repeated three times then quenched with Quenching buffer (50 mM ethanolamine in PBS) at room temperature for 5-minutes. Excess antibody (unlinked) was removed using two 5-minute washes with Elution buffer (1 M glycine pH 3), and the remaining antibody-bead complex was rotated overnight at 4°C in CLIP lysis buffer to be used the following day.

##### Cell lysis, immunoprecipitation and library construction

UV irradiated cell pellets were processed as previously described by Van Nostrand et al., 2017 in biological duplicate. Briefly, cross-linked cell pellets were lysed with 0.5mL Lysis buffer (50 mM Tris-HCl, 100 mM NaCl, 1% NP-40, 0.1% SDS, 0.5% sodium deoxycholate, 2μg/mL aprotinin, 2μg/mL leupeptin, 0.7μg/mL pepstatin, pH 7.4) for 10 minutes on ice followed by ultrasonication (Bioruptor). 2μl of Turbo DNase (LifeTech) and 5 μl 1:25 diluted RNase I (LifeTech) was added to lysates respectively, then incubated at 37°C for 5 minutes exactly before adding 30ml RNase inhibitor (NEB). Lysates were pelleted and antibody-bead complexes were added to the supernatant followed by 1-hour rocking at 4°C to capture ZFP36-RNA complexes. Next, 2% of total material from each immunoprecipitation was saved as input fractions, while the remaining IP fraction was stringently washed and ligated with 3’ barcoded linkers. 20% of the input or IP fractions were then separated by SDS-PAGE and immunoblotted for ZFP36 and GAPDH for evidence of successful processing and to provide a reference for ZFP36-RNA complex excision. The remaining 80% of input or IP fractions was similarly separated by SDS-PAGE and transferred to a nitrocellulose membrane, excising 75kD above the molecular weight of ZFP36 corresponding to approximately 45–120kD. RNA was then released from the membrane with Proteinase K (NEB) and purified with acid-phenol:chloroform, pH 4.5 (with IAA, 125:24:1) (Invitrogen Ambion #AM9722). Samples were concentrated (Zymo) followed by ligation of a 3’ liker to SMInput samples. Next, all samples were reverse transcribed and depleted of RNA contamination before ligating a random-mer adapter to the 5’ ends. Libraries were quantified by qPCR using primers that anneal to adapter sequences flanking each molecule of RNA. The PCR cycle number for final eCLIP library amplification was two less than the RT-qPCR Ct values. Replicate libraries were independently sequenced (paired-end; 2×100bp) on the NOVASeq 6000 platform.

##### eCLIP-seq data processing and analysis

Reads were processed and aligned to the mouse mm10 genome. UMIs were extracted using umi_tools, adapters were removed with cutadapt, then reads were aligned with STAR and deduplicated with umi_tools.^62–64^ Peaks were called with pureclip, using a size-matched input control for each CLIP library.^65^ To identify ZFP36-specific peaks, peaks were identified in both the ZFP36/L1/L2 wildtype (TFWT) and triple knockout (TFKO.1) MEF conditions for each library; peaks identified in TFKO.1 libraries were excluded from all downstream TFWT analyses. Motif analysis was performed in a peak-wise manner with Homer.^66^ Binding scores were summed across each gene to obtain a per-gene ZFP36 binding score. For gene ontology analysis, genes with a score >0 in both replicates were used with clusterProfiler to find overrepresented categories.^67^

#### ZFP36 CLIP-qPCR

ZFP36/L1/L2 wildtype (TFWT) or triple knockout (TFKO.1) MEFs were serum deprived overnight, stimulated for 40-minutes with 10% FBS, then UV-irradiated, snap frozen, and stored at −80°C as described for eCLIP-seq methods outlined above. At time of processing, cells were lysed with 0.5mL CLIP Lysis buffer (50 mM Tris-HCl, 100 mM NaCl, 1% NP-40, 0.1% SDS, 0.5% sodium deoxycholate, 2μg/mL aprotinin, 2μg/mL leupeptin, 0.7μg/mL pepstatin, pH 7.4) for 10 minutes on ice followed by 5-minutes DNase digestion at 37°C. Importantly, RNase inhibitor was added to the lysates and no RNase digestion step was performed to ensure recovery of full-length transcripts in complex with ZFP36. Lysates were then pelleted and the supernatant was collected followed by protein quantification using a BCA assay. Equal amounts of protein from TFWT or TFKO.1 MEF conditions were mixed with 25ug anti-ZFP36 antibody (Millipore ABE285) pre-conjugated to 100ul Protein A Dynabeads respectively, and rocked for 1-hour at 4°C. On-bead Proteinase K (NEB) digestion was performed to release RNA, which was then purified with acid-phenol:chloroform, pH 4.5 (with IAA, 125:24:1) and concentrated to 20ul final volume (Zymo). Next, 10ul of isolated RNA was used for cDNA synthesis (Biorad iScript) in 20ul reactions then diluted 5-fold in nuclease-free water. For each qPCR reaction, 2ul of diluted cDNA combined with 0.5μM primers in 20μl Power SYBR master mix (Applied Biosystems) was amplified using a QuantStudio5 (Applied Biosystems). Relative ZFP36 binding enrichment to target mRNAs over background was calculated according to previously described methods deriving ΔΔCt with Rplpo as the reference gene for IP samples.^68^ Tuba1b was used as a negative control, Ptgs2 served as a positive control. Data are presented as ZFP36 target binding enrichment fold change relative to TFKO.1 cells; signal from TFKO.1 conditions is independent of ZFP36. (Table 1 provides primer sequences used).

#### ENO2 3′UTR luciferase reporter analysis

The Eno2 3′UTR was amplified from a commercially available ENO2 expression plasmid (Origene MC201508) and cloned into a firefly luciferase reporter construct (Origene PS100062). Site-directed mutagenesis was used to delete the AREs found bound by ZFP36 in our eCLIP-seq dataset, generating a mutant 3′UTR reporter construct (Δ ARE) (primers listed in Table 2). 1×10^4^ HeLa cells were seeded per well in a 96-well dish then, the following day, co-transfected with CMV-driven ZFP36 (6ng/well) or ZFP36^C124R^ mutant deficient in RNA-binding (6ng/well), firefly luciferase reporter construct (50ng/well of full length or Δ ARE), and Renilla luciferase reporter (50ng/well) using PolyFect Transfection Reagent (Qiagen) (n = 6 wells per condition).^69^ After a 24-h incubation, the transfection mixture was replaced with fresh media and luminescence was measured the following day using Promega DualGlo Luciferase Assay System (Promega E2920) according to the manufacturer’s instructions. Firefly luciferase activity was normalized to Renilla luciferase activity in each well to account for transfection efficiency. Data are expressed as fold change relative to full length Eno2 3′UTR reporter in the presence of CMV-driven ZFP36 capable of binding RNA.

#### Analysis of human fetal atlas data

Fetal human single cell ATAC and RNA-seq data of 15 organs was downloaded from DESCARTES.^18,40^ Normalized gene expression (TPM) per organ and normalized bigwigs of chromatin accessibility per cell type was used. To summarize chromatin accessibility per tissue, the normalized bigwig score across each promoter (+/−1kb TSS) was summed for each cell type, then the promoter accessibility scores for each gene were averaged across the cell types in each organ. This resulted in a single gene expression and promoter accessibility score for each gene in each of the 15 organs.

#### RNA sequencing library construction and analysis

Total RNA was isolated using Quiagen RNeasy Kit with the inclusion of an on-column DNase treatment. Library construction was performed by UCLA Jonsson Comprehensive Cancer Center Genomics Core. Final libraries were single-read sequenced using the Illumina HiSeq 3000 with a 50 base pair read length and a depth of 25 million reads per sample. FASTQ files were aligned to the mouse genome (mm10) using STAR. DESeq2 was used to quantify transcript abundance and differential expression. Plots and heatmaps were generated in R using ComplexHeatmap and Graphics packages.

#### Mouse retina experiments

##### Retina immunostaining

At P6, post-enucleation, whole eyes were fixed directly in 4% (wt/vol) PFA-PBS for 15min, followed by retinal dissection in 2% (wt/vol) PFA-PBS at a total fixation time of 1h in 2% PFA. Retinas were washed 3 times for 5 min in 1xPBS before blocking for 1h at room temperature. Primary antibodies were incubated overnight at 4°C in blocking buffer. The following day, retinas were washed 3 times for 5 min in 1xPBS before secondary antibodies were applied for 1h at room temperature. Retinas were washed 3 times for 5 min in 1xPBS and flat-mounted on slides in ProLong Gold Antifade Mountant (Fisher Scientific #P36930). Antibodies and their respective dilutions used for immunostaining are as follows: CD31 (1:200; AB_396660), ENO2 (1:100; AB_1619729), Donkey anti-mouse a568 (1:400; ab175700), and Donkey anti-rat a488 (AB_2535794).

##### Retina imaging and analysis

Imaging was performed using A1R HD25 confocal microscope (Nikon). Z-stack and tile scan features were used to image the entire retinal surface and superficial plexus. Tiles were stitched into a single large image (NIS-Elements, Nikon). Quantification was performed on stitched images using Imaris software (Imaris 9.9.0, Bitplane). For quantification of ENO2 staining, mean fluorescent intensity was calculated within 3D surface rendered from CD31 staining. For figure images, Denoise.AI (Nikon) was used to remove Poisson shot noise. Images were acquired using 20x objective.

### QUANTIFICATION AND STATISTICAL ANALYSES

All experiments were carried out in biological triplicate unless otherwise indicated; eCLIP-seq was performed in biological duplicate and a linear regression was used to analyze the scatterplot of cross-link scores. We performed a two-way ANOVA with Sidak’s multiple comparison test to compare Zfp36/l1/l2 mRNA levels by RT-qPCR in response to growth factor stimulation or inhibitor treatments. Bar graphs depict individual data points compared by a two-tailed Student’s t test. All significant results were defined as having a p-value < 0.05. Asterisks indicate the significance of the p-value: *p < 0.05; **p < 0.01; ***p < 0.001.

## Supplementary Material

1

## Figures and Tables

**Table 1.** RT-PCR primers

Gene	Forward (5′ to 3′)	Reverse (5′ to 3′)	Species
Zfp36	AACGGAACTCTGCCACAAGT	AGTGGCATCGAGAGCCATAG	mouse
Zfp36l1	TTCACGACACACCAGATCCT	TGAGCATCTTGTTACCCTTGC	mouse
Zfp36l2	TTCTACGATATCGACTTCTTGTGC	AAGCCCGGAGTGAAGCTC	mouse
Eno2	AGGTAGCAGATCAGGGGAGG	GGATCCCACATCACCAGCAA	mouse
Ptgs2	TGAGTACCGCAAACGCTTCT	CAGCCATTTCCTTCTCTCCTGT	mouse
Fgf21	CCTTGAAGCCAGGGGTCATT	AGGATCAAAGTGAGGCGATCC	mouse
Rplpo	CACTGGTCTAGGACCCGAGAAG	GGTGCCTCTGGAGATTTTCG	mouse
Tuba1b	GAGACCCGGTGTCTGCTTC	GAGATGCACTCACGCATGATA	mouse
Slc4a7	GAGTCGGATTCCCCTGGTTC	TCCTTTCAAGCAAGTGAGGGT	mouse
Zfp36	GACTGAGCTATGTCGGACCTT	GAGTTCCGTCTTGTATTTGGGG	human
Zfp36l1	GATGACCACCACCCTCGT	TGGGAGCACTATAGTTGAGCATC	human
Zfp36l2	CTGCTGCTGACTGCGGTA	ATCCAGACCCACAACTTTGC	human
Rplpo	TCTACAACCCTGAAGTGCTTGAT	CAATCTGCAGACAGACACTGG	human

**Table 2.** Site-directed mutagenesis PCR primers

Site	Forward (5′ to 3′)	Reverse (5′ to 3′)
ZFP36 C124R	CGCTACGGGGCC AAGCGCCAGTTT GCCCATGG	CCATGGGCAAA CTGGCGCTTGG CCCCGTAGCG
Eno2 ΔARE	TACATACACA AGCACAGCTCA GCTGGTCAGTCAG	CTGACTGACCA GCTGAGCTGTG CTTGTGTATGTA

**Table:** KEY RESOURCES TABLE

REAGENT or RESOURCE	SOURCE	IDENTIFIER
Antibodies		
Tristetraprolin	Millipore	ABE285
Tristetraprolin	Cell Signaling Technology	Cat# 71632; RRID:AB_2799806
BRF1/2	Cell Signaling Technology	Cat# 2119; RRID:AB_10695874
ERK	Cell Signaling Technology	Cat# 9102; RRID:AB_330744
phospho-Thr202/Tyr204 ERK	Cell Signaling Technology	Cat# 4370; RRID:AB_2315112
S6 kinase	Cell Signaling Technology	Cat# 2708; RRID:AB_390722
phospho-T389 S6 kinase	Cell Signaling Technology	Cat# 9234; RRID:AB_2269803
β-actin	Cell Signaling Technology	Cat# 3700; RRID:AB_2242334
c-Myc	Cell Signaling Technology	Cat# 18583; RRID:AB_2895543
GAPDH	Cell Signaling Technology	Cat# 5174; RRID:AB_10622025
α-tubulin	Sigma	Cat# T6074; RRID:AB_477582
Recombinant Anti-Heme Oxygenase 1 antibody	Abcam	Cat# ab68477
CD31	BD Biosciences	Cat# 557355; RRID:AB_396660
Donkey Anti-Mouse IgG H&L (Alexa Fluor^®^ 568) preadsorbed	Abcam	Cat# ab175700
Donkey anti-Rat IgG (H + L) Highly Cross-Adsorbed Secondary Antibody, Alexa Fluor 488	Thermo Fisher Scientific	Cat# A-21208; RRID:AB_2535794
ENO2	ABFrontier	Cat# LF-MA0097

Bacterial and virus strains		

Human Adenovirus Type5 (dE1/E3)	Vector Biolabs	Cat. # 1045/1060

Chemicals, peptides, and recombinant proteins		

Acid-Phenol:Chloroform, pH 4.5 (with IAA, 125:24:1)	Thermo Fisher Scientific	Cat# AM9720
Proteinase K	NEB	Cat# P8107S
Lipofectamine 3000	Thermo Fisher Scientific	Cat# L3000001
RNase inhibitor	NEB	M0314S
RNase I	Thermo Fisher Scientific	EN0601
Turbo DNase	Thermo Fisher Scientific	AM2238
U-13C6-glucose	Cambridge Isotope Laboratories	Cat# CLM-1396–1
Polybrene	Santa Cruz	Cat# sc-134220
PolyFect Transfection Reagent	Qiagen	Cat# 220002–078
Blasticidin	Invivogen	Cat# ant-bl-1
PD 0325901	Sigma	Cat# PZ0162
SB 203580	Sigma	Cat# S8307
Torin-1	Tocris	Cat# 4247
Cyclohexamide	Sigma	Cat# 01810
Actinomycin D	Sigma	Cat# A1410
Recombinant Human EGF Protein	Thermo Fisher Scientific	Cat# PHG0313
Recombinant Human FGF-basic	Peprotech	Cat# 100–18B
Human recombinant insulin	Thermo Fisher Scientific	Cat# 12585014
Recombinant Human IGF-I	Peprotech	Cat# 100–11
Recombinant Human PDGF-BB	Peprotech	Cat# 100–14B
ProLong Gold Antifade Mountant	Thermo Fisher Scientific	Cat# P36930
Dynabeads^™^ Protein A	Thermo Fisher Scientific	Cat# 10001D

Critical commercial assays		

Promega DualGlo Luciferase Assay System	Promega	Cat# E2920
iScript^™^ Reverse Transcription Supermix	Bio Rad	Cat# 1708841
Pierce^™^ BCA Protein Assay Kit	Thermo Fisher Scientific	Cat# 23227
ΔRNA Clean & Concentrator-5	Zymo Research	Cat# R1013
Q5 PCR Master Mix	NEB	Cat# M0492S
AffinityScript reverse transcriptase	Agilent Technologies	Cat# 600107
T4 PNK	NEB	Cat# M0201L
Applied Biosystems^™^ Power SYBR^™^ Green PCR Master Mix	Applied Biosystems	Cat# 4368706

Deposited data		

RNA-seq	NCBI Sequence Read Archive	SRA: PRJNA942602
eCLIP-seq	NCBI Sequence Read Archive	SRA: PRJNA943291

Experimental models: Cell lines		

A549 (human lung adenocarcinoma)	Steven Dubinett (University of California, Los Angeles)	RRID: CVCL_0023
HeLa (human cervical adenocarcinoma)	Steven Bensinger (University of California, Los Angeles)	RRID: CVCL_0030
MCF-10A (human breast epithelial)	Frank McCormick (University of California, San Francisco)	RRID: CVCL_0598
293T (human embryonic kidney)	Steven Bensinger (University of California, Los Angeles)	RRID: CVCL_0063
HUVECs	M. Luisa Iruela-Arisp (University of California, Los Angeles)	RRID:CVCL_2959
HFF-1	ATCC	RRID:CVCL_3285
Zfp36/l1/l2 triple-floxed MEFs (mouse embryonic fibroblasts)	Martin Turner (The Babraham Institute, Cambridge)	Hodson et al., 2010; New et al., 2017.

Experimental models: Organisms/strains		

Mouse strain: C57BL/6J	The Jackson Laboratory	Cat# 000664
Mouse strain: Tg(Cdh5-cre/ERT2)-Zfp36flox/flox Gt(ROSA)26Sor tm14(CAG tdTomato)Hze)	M. Luisa Iruela-Arisp (Northwestern University)	Sorensen et al., 2009; Qiu, L. Q. et al., 2012; Lizama et al., 2015.

Oligonucleotides		

sgRNA targeting ROSA26: GTAGTAATGAGAGTTCACTG	This paper	N/A
sgRNA targeting mouse Eno2 #1: ATAGAGATCCACCTCCACGG	This paper	N/A
sgRNA targeting mouse Eno2 #2: GATTTGGGCCCGAGAGATCT	This paper	N/A
qPCR primers (Table 1)	NCBI Primer-BLAST	https://www.ncbi.nlm.nih.gov/tools/primer-blast/
Site directed mutagenesis primers (Table 2)	Agilent QuickChange II primer design	https://www.agilent.com/store/primerDesignProgram.jsp

Recombinant DNA		

PCMV6-Kan/Neo-Mouse Eno2	Origene	Cat# MC201508
pMirTarget vector	Origene	Cat# PS100062
CMV-ZFP36	This paper	N/A
Renilla Luciferase	William Lowry (University of California, Los Angeles)	Cinkornpumin et al., 2017.
lentiCRISPR v2-Blast	Addgene	Cat# 83480
eGFP Adenovirus	Vector Biolabs	Cat# 1060
Cre Recombinase Adenovirus	Vector Biolabs	Cat# 1045

Software and algorithms		

Prism8	GraphPad	https://www.graphpad.com/scientificsoftware/prism/
Image J	NIH	https://imagej.nih.gov/ij/
Imaris 9.9.0	GitHub	https://github.com/topics/imaris
MZmine 2	GitHub	http://mzmine.github.io/
AccuCor	GitHub	https://github.com/lparsons/accucor
STAR	GitHub	https://github.com/alexdobin/STAR
DESeq2	GitHub	https://github.com/mikelove/DESeq2
UMI-tools	GitHub	https://github.com/CGATOxford/UMI-tools
PureCLIP	GitHub	https://github.com/skrakau/PureCLIP
clusterProfiler	GitHub	https://github.com/YuLab-SMU/clusterProfiler

Other		

QuantStudio5	Thermo Fisher Scientific	N/A
Z1 Particle Counter	Beckman Coulter	N/A
A1R HD25 confocal microscope	Nikon	N/A
NOVASeq 6000	Illumina	N/A
Bioruptor	Diagenode	UCD-200
Q Exactive	Thermo Fisher Scientific	N/A

## Highlights

- Growth factor-induced MAPK/mTOR signaling leads to ZFP36 expression in cells and tissues
- ZFP36 directly binds metabolic enzyme and transporter mRNAs from diverse pathways
- ZFP36 regulates expression of the glycolytic enzyme Enolase 2 through mRNA decay
- Cellular metabolism is dysregulated upon ZFP36 loss
