# Supplementary material for: ZFP36-mediated mRNA decay regulates metabolism
Source: Cell Rep. Author manuscript; Available in PMC 2023 Jul 10. (PMC10332406; doi:10.1016/j.celrep.2023.112411)
Supplement: 1 [file NIHMS1905349-supplement-1.pdf]

Cell Reports, Volume *42*

Supplemental information

ZFP36-mediated mRNA decay regulates metabolism

Andrew C. Cicchetto, Elsie C. Jacobson, Hannah Sunshine, Blake R. Wilde, Abigail S. Krall, Kelsey E. Jarrett, Leslie Sedgeman, Martin Turner, Kathrin Plath, M. Luisa Iruela-Arispe, Thomas Q. de Aguiar Vallim, and Heather R. Christofk

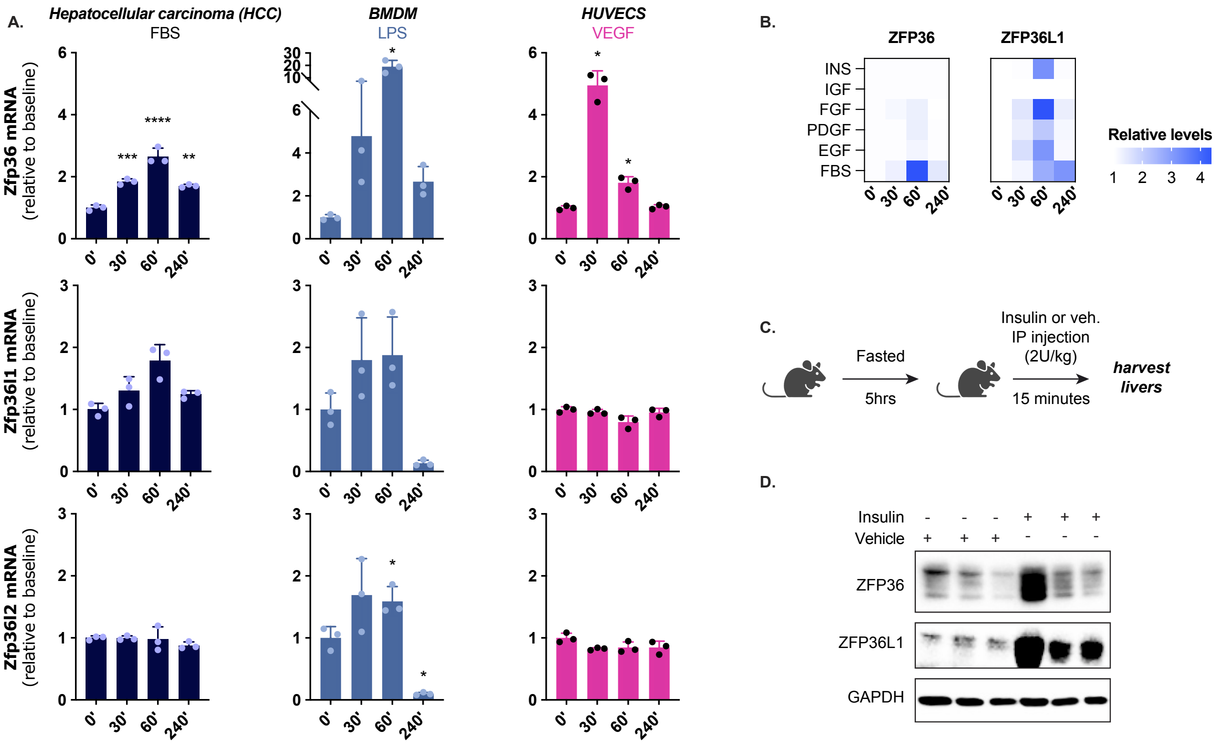

A.
Hepatocellular carcinoma (HCC)
FBS
BMDM
LPS
HUVECS
VEGF
Zfp36 mRNA
(relative to baseline)
Zfp36l1 mRNA
(relative to baseline)
Zfp36l2 mRNA
(relative to baseline)
0' 30' 60' 240'
B.
ZFP36
ZFP36L1
INS
IGF
FGF
PDGF
EGF
FBS
Relative levels
1 2 3 4
C.
Fasted
5hrs
Insulin or veh.
IP injection
(2U/kg)
15 minutes
harvest livers
D.
Insulin - - - + + +
Vehicle + + + - - -
ZFP36
ZFP36L1
GAPDH

Figure S1: Related to Figure 1. ZFP36 family member induction is stimulus- and tissue-specific. (A) Relative Zfp36/l1/l2 transcript levels of hepatocellular carcinoma cells stimulated with 10% FBS, bone marrow-derived macrophages stimulated with LPS, and human umbilical vein endothelial cells stimulated with VEGF. All stimulations were performed for 0, 30, 60 or 240-minutes following overnight serum deprivation. **(B)** Quantification of ZFP36 and ZFP36L1 immunoblots presented in Figure 1B using Image J software. Data is normalized to tubulin and presented relative to t = 0 for each respective growth factor. **(C)** Schematic of intraperitoneal (IP) insulin injection for 15 minutes in fasted C57BL/6 mice. **(D)** 15-minute vehicle or insulin-stimulated mouse lysates immunoblotted for ZFP36, ZFP36L1 and GAPDH. All experiments were performed with biological replicates. Error bars denote SD (n=3). *p<0.05; **p<0.01; ***p< 0.001. A 2way ANOVA Dunnett's multiple comparisons test was performed for (A).

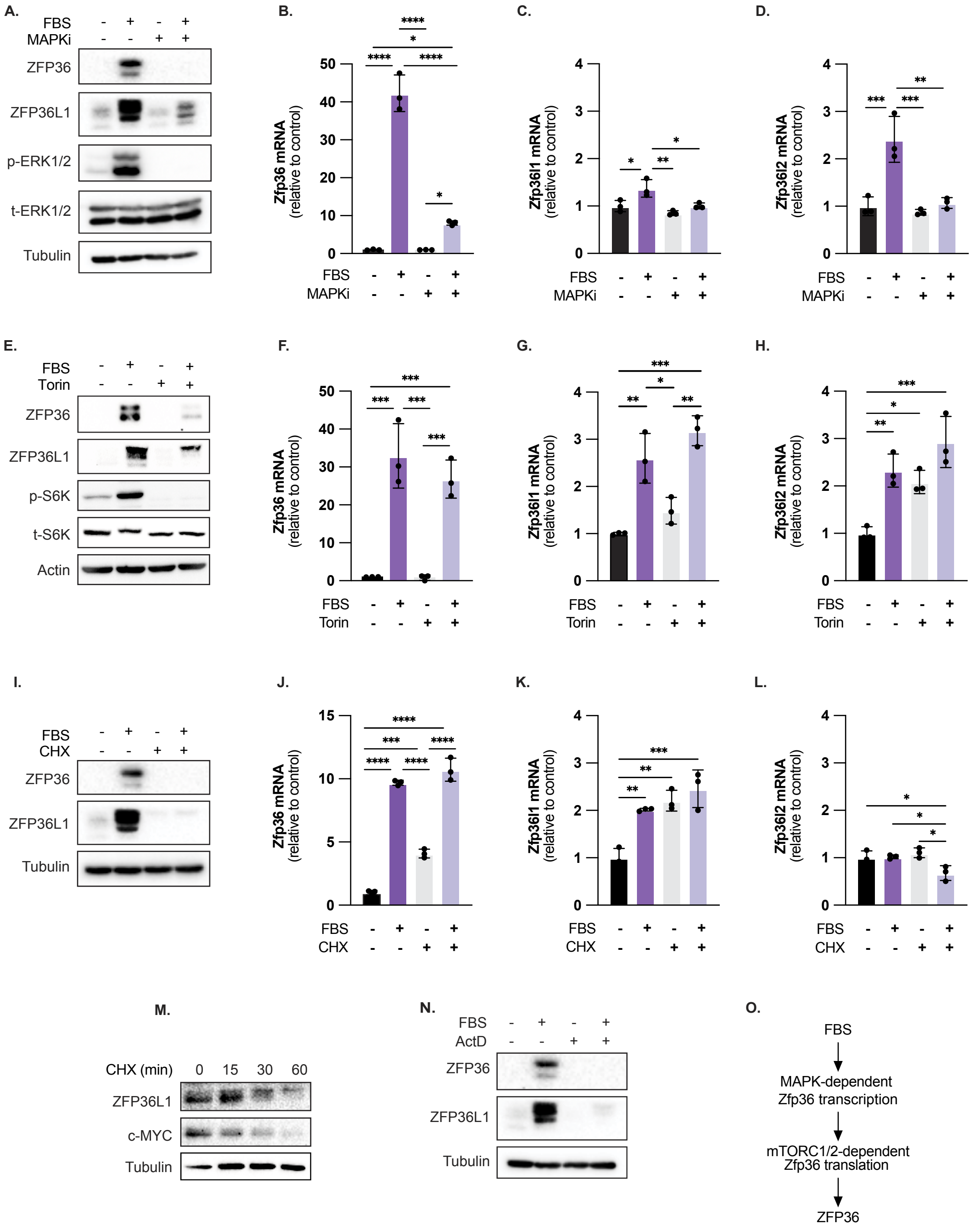
A.
FBS
MAPKi
ZFP36
ZFP36L1
p-ERK1/2
t-ERK1/2
Tubulin
B.
Zfp36 mRNA (relative to control)
FBS
MAPKi
C.
Zfp36l1 mRNA (relative to control)
D.
Zfp36l2 mRNA (relative to control)
E.
Torin
p-S6K
t-S6K
Actin
F.
G.
H.
I.
CHX
J.
K.
L.
M.
CHX (min) 0 15 30 60
c-MYC
N.
ActD
O.
FBS
MAPK-dependent Zfp36 transcription
mTORC1/2-dependent Zfp36 translation
ZFP36

Figure S2: Related to Figure 1. ZFP36 proteins are immediate early genes downstream of MAPK and mTORC1/2 signaling. (A) Immunoblot of MEF lysates for ZFP36, ZFP36L1, p42/44 MAPK (total and Thr202/Tyr204), and tubulin. Following overnight serum-deprivation, cells were pretreated for 15-minutes with a small molecule inhibitor cocktail (MAPKi) containing p38 inhibitor SB2035800 (1μM) + Mek1/2 inhibitor PD0325901 (1μM) or DMSO control, +/- 1-hour 10% FBS. **(B-D)** Relative MEF transcript levels of Zfp36 (B), Zfp36l1 (C), and Zfp36l2 (D) +/- 30-minute 10% FBS in the presence or absence of MAPKi pretreatments as described in A. **(E)** Immunoblot of MEF lysates for ZFP36, ZFP36L1, S6K (total and phospho-Thr389) and actin. Cells were serum-deprived overnight and pretreated for 15-minutes with mTORC1/2 inhibitor (Torin, 250nM) or DMSO control, +/- 1 hour 10% FBS. **(F-H)** Relative MEF transcript levels of Zfp36 (F), Zfp36l1 (G), and Zfp36l2 (H) +/- 30-minute 10% FBS in the presence of Torin or DMSO pretreatment as described in (E). **(I)** Immunoblot of MEF lysates for ZFP36, ZFP36L1 and tubulin. Cells were serum-deprived overnight followed by a 15-minute pretreatment with the translation elongation inhibitor cycloheximide (CHX, 100μg/mL) or DMSO control, +/- 1 hour 10% FBS. **(J-L)** Relative MEF transcript levels of Zfp36 (J), Zfp36l1 (K), and Zfp36l2 (L) +/- 30-minute 10% FBS in the presence of CHX or DMSO control as described in I. **(M)** Immunoblot of MEF lysates treated with DMSO or 100mg/mL CHX for 15, 30, or 60-minutes. Lysates were immunoblotted for ZFP36L1, c-MYC and tubulin. Cells were maintained in regular growth medium without being acutely stimulated with serum and therefore lack ZFP36 signal. **(N)** Immunoblot of MEF lysates serum-deprived overnight +/- 1-hour 10% FBS in the presence of the transcription inhibitor actinomycin D (ActD, 5μg/mL) or DMSO control. Lysates were immunoblotted for ZFP36, ZFP36L1, and tubulin. **(O)** Model of acute growth factor signaling-dependent regulation of ZFP36 expression. All experiments were performed with biological replicates. Error bars denote SD (n=3). *p<0.05; **p<0.01; ***p< 0.001.

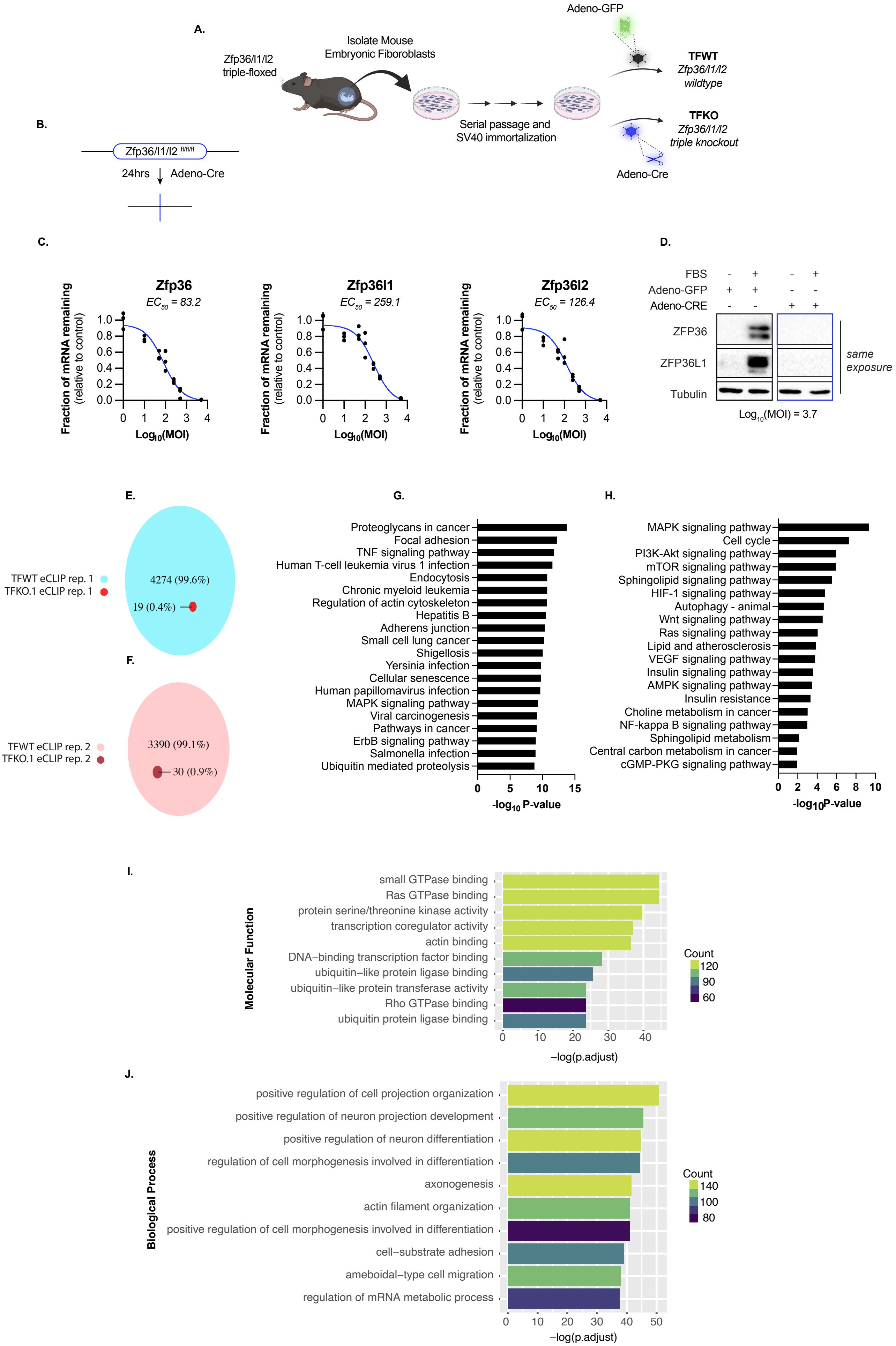

A.
Zfp36/l1/l2
triple-floxed
Isolate Mouse
Embryonic Fibroblasts
Serial passage and
SV40 immortalization
Adeno-GFP
TFWT
Zfp36/l1/l2
wildtype
TFKO
Zfp36/l1/l2
triple knockout
Adeno-Cre
B.
Zfp36/l1/l2 fl/fl/fl
24hrs
Adeno-Cre
C.
Zfp36
EC50 = 83.2
Zfp36l1
EC50 = 259.1
Zfp36l2
EC50 = 126.4
Fraction of mRNA remaining
(relative to control)
Log10(MOI)
D.
FBS
Adeno-GFP
Adeno-CRE
ZFP36
ZFP36L1
Tubulin
same
exposure
Log10(MOI) = 3.7
E.
TFWT eCLIP rep. 1
TFKO.1 eCLIP rep. 1
4274 (99.6%)
19 (0.4%)
F.
TFWT eCLIP rep. 2
TFKO.1 eCLIP rep. 2
3390 (99.1%)
30 (0.9%)
G.
Proteoglycans in cancer
Focal adhesion
TNF signaling pathway
Human T-cell leukemia virus 1 infection
Endocytosis
Chronic myeloid leukemia
Regulation of actin cytoskeleton
Hepatitis B
Adherens junction
Small cell lung cancer
Shigellosis
Yersinia infection
Cellular senescence
Human papillomavirus infection
MAPK signaling pathway
Viral carcinogenesis
Pathways in cancer
ErbB signaling pathway
Salmonella infection
Ubiquitin mediated proteolysis
-log10 P-value
H.
MAPK signaling pathway
Cell cycle
PI3K-Akt signaling pathway
mTOR signaling pathway
Sphingolipid signaling pathway
HIF-1 signaling pathway
Autophagy - animal
Wnt signaling pathway
Ras signaling pathway
Lipid and atherosclerosis
VEGF signaling pathway
Insulin signaling pathway
AMPK signaling pathway
Insulin resistance
Choline metabolism in cancer
NF-kappa B signaling pathway
Sphingolipid metabolism
Central carbon metabolism in cancer
cGMP-PKG signaling pathway
-log10P-value
I.
Molecular Function
small GTPase binding
Ras GTPase binding
protein serine/threonine kinase activity
transcription coregulator activity
actin binding
DNA−binding transcription factor binding
ubiquitin−like protein ligase binding
ubiquitin−like protein transferase activity
Rho GTPase binding
ubiquitin protein ligase binding
Count
120
90
60
−log(p.adjust)
J.
Biological Process
positive regulation of cell projection organization
positive regulation of neuron projection development
positive regulation of neuron differentiation
regulation of cell morphogenesis involved in differentiation
axonogenesis
actin filament organization
positive regulation of cell morphogenesis involved in differentiation
cell−substrate adhesion
ameboidal−type cell migration
regulation of mRNA metabolic process
Count
140
100
80
−log(p.adjust)

Figure S3: Related to Figure 2. ZFP36 eCLIP-seq replicates are highly reproducible revealing known and novel ZFP36 targets. (A) Schematic diagramming Zfp36/l1/l2 triple-floxed MEF cell line generation and adenoviral-based approach for *in vitro* Cre recombinase delivery (adeno-Cre), or GFP control (adeno-GFP), to derive Zfp36/l1/l2 triple-foxed wildtype (TFWT) and triple-floxed knockout (TFKO) cells from an isogenic cell population. **(B)** Schematic diagramming Cre recombinase-mediated recombination of target Lox-P sites flanking both alleles of each of the three ZFP36 family members. **(C)** Relative Zfp36/l1/l2 transcript levels of MEFs pretreated with increasing amounts of adeno-Cre recombinase in serum-free medium for 24-hours, then stimulated with 10% FBS for 30-minutes. Data is fit to an inhibitory EC50 model. **(D)** Immunoblot of triple-floxed MEFs pretreated with Adeno-GFP/CRE (Log_{10}MOI = 3.7) for 24hrs in serum-free medium then stimulated with 10% FBS for 1-hour. Lysates were immunoblotted for ZFP36, ZFP36L1 and tubulin. **(E, F)** eCLIP-seq replicates 1 (E) and 2 (F) depicting number of unique mRNAs bound in ZFP36/L1/L2 wildtype (TFWT) or triple knockout clone (TFKO.1) MEFs. **(G, H)** Unbiased top 20 (G) or signaling and metabolism-related (H) KEGG pathway overrepresentation. Analysis includes common mRNAs identified in both eCLIP-seq replicates, excluding mRNAs identified in ZFP36/L1/L2 triple knockout (TFKO.1) replicates. **(I, J)** GO Terms: Molecular Function (I) or Biological Process (J) of ZFP36-bound mRNAs identified by eCLIP-seq analysis.

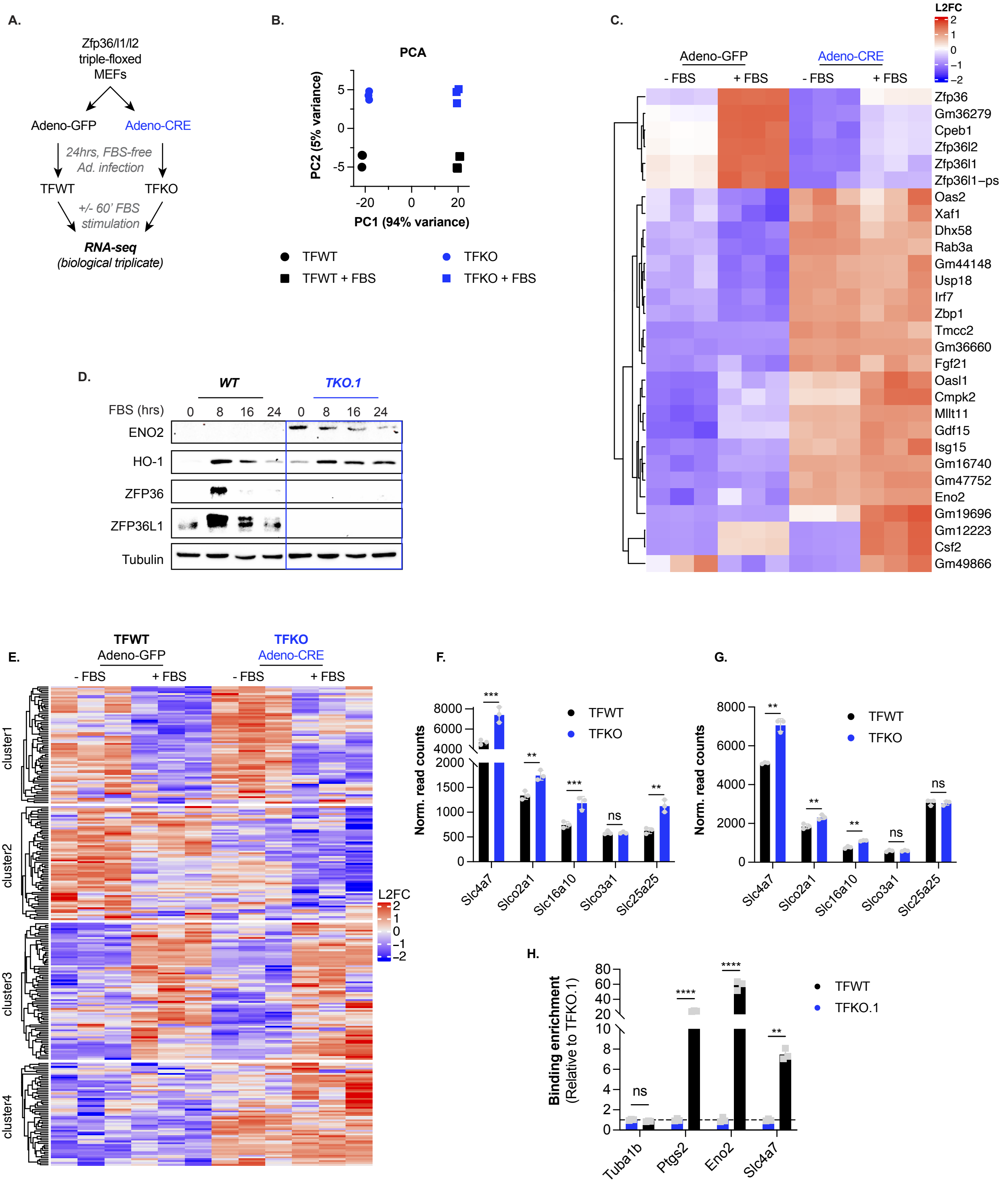
A.
Zfp36/l1/l2 triple-floxed MEFs
Adeno-GFP
Adeno-CRE
24hrs, FBS-free Ad. infection
TFWT
TFKO
+/- 60' FBS stimulation
RNA-seq (biological triplicate)
B.
PCA
PC2 (5% variance)
PC1 (94% variance)
TFWT
TFKO
TFWT + FBS
TFKO + FBS
C.
L2FC
Adeno-GFP
Adeno-CRE
- FBS
+ FBS
Zfp36
Gm36279
Cpeb1
Zfp36l2
Zfp36l1
Zfp36l1−ps
Oas2
Xaf1
Dhx58
Rab3a
Gm44148
Usp18
Irf7
Zbp1
Tmcc2
Gm36660
Fgf21
Oasl1
Cmpk2
Mllt11
Gdf15
Isg15
Gm16740
Gm47752
Eno2
Gm19696
Gm12223
Csf2
Gm49866
D.
WT
TKO.1
FBS (hrs)
0 8 16 24 0 8 16 24
ENO2
HO-1
ZFP36
ZFP36L1
Tubulin
E.
TFWT
Adeno-GFP
TFKO
Adeno-CRE
- FBS
+ FBS
cluster1
cluster2
cluster3
cluster4
L2FC
F.
Norm. read counts
TFWT
TFKO
Slc4a7
Slco2a1
Slc16a10
Slco3a1
Slc25a25
G.
Norm. read counts
TFWT
TFKO
H.
Binding enrichment (Relative to TFKO.1)
TFWT
TFKO.1
Tuba1b
Ptgs2
Eno2
Slc4a7

Figure S4: Related to Figure 3. RNA-seq identifies ZFP36/L1/L2-dependent gene expression in acute growth factor signaling context. (A) Schematic diagramming adenoviral-based acute Zfp36/l1/l2 family member gene loss approach for performing RNA-seq. Zfp36/l1/l2 triple-floxed MEFs were exposed to adeno-GFP (TFWT) or adeno-Cre (TFKO) (Log_{10}(MOI) = 3.7) for 24-hours in serum-free medium, +/- one hour 10% FBS stimulation, followed by RNA isolation and sequencing. **(B)** Principal component analysis; PC1 vs PC2 of RNA-seq results for TFWT or TFKO MEFs serum-deprived or 10% FBS-stimulated conditions. **(C)** Unbiased hierarchical clustering of top DEGs (p-value of ≤ 0.01 and a log2(fold-change) of ≥1 or ≤-1), in serum-deprived or -stimulated RNA-seq conditions as described in A. **(D)** Immunoblot of ZFP36/L1/L2 triple-floxed wildtype (TFWT) MEFs or a triple knockout clone (TFKO.1) serum-deprived overnight then stimulated with 10% FBS for 8, 16, or 24 hours. Lysates were immunoblotted for ENO2, HO-1, ZFP36, ZFP36L1 and Tubulin. **(E)** Unbiased hierarchical clustering of all solute carrier (SLC) mRNAs from RNA-seq experiment, without applying statistical cut-off filters. **(F, G)** Normalized RNA-seq read counts of top 5 eCLIP-seq scoring ZFP36-bound SLC mRNAs in serum-deprived (F) or serum-stimulated (G) conditions. **(H)** ZFP36 CLIP-qPCR of indicated genes. TFWT or TFKO.1 MEFs were serum starved overnight then stimulated for 40-minutes with 10% FBS prior to cross-linking. RNA was isolated from immunoprecipitated ZFP36 and RT-qPCR was performed. Data is presented as binding enrichment fold change relative to TFKO.1 conditions.

A.

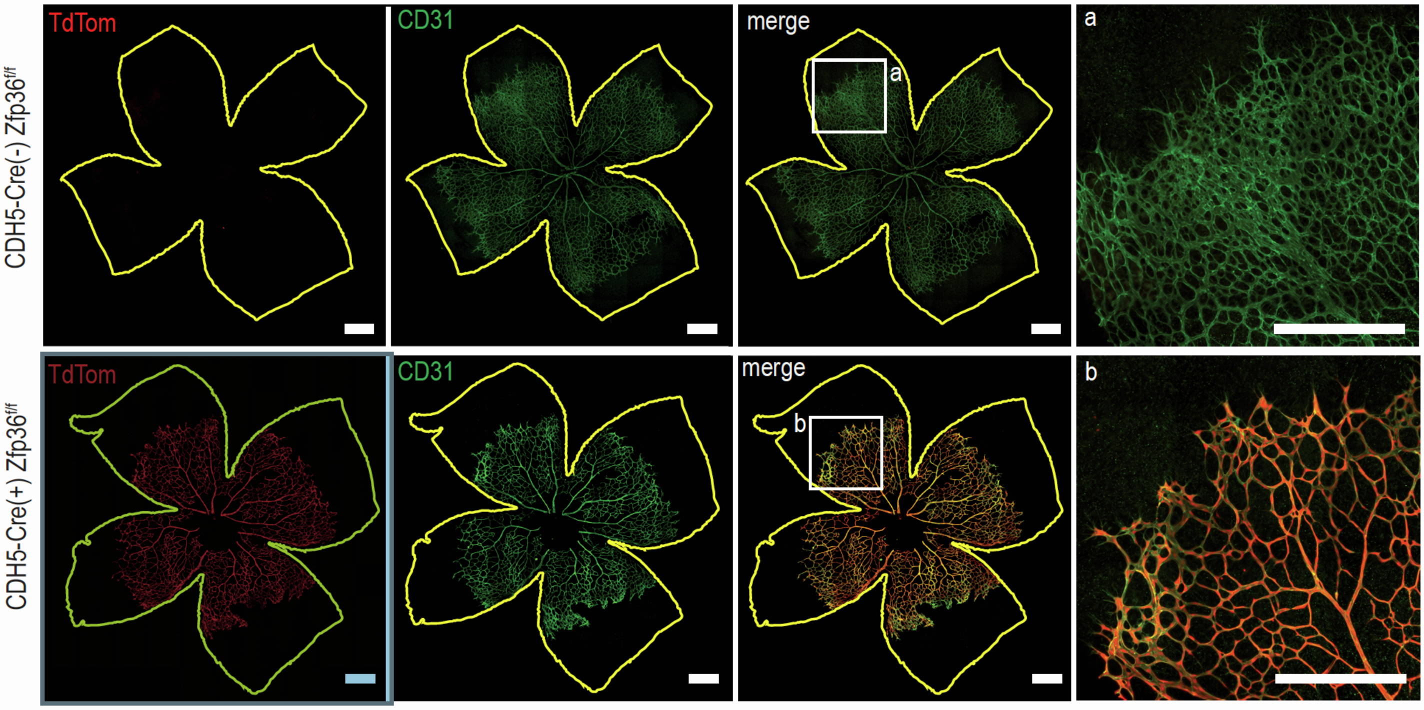

scale bar 400um

Figure S5: Related to Figure 5. TdTomato reporter for CDH5-driven Cre recombination of Zfp36 alleles.

(A) Cre(-) or Cre(+) images corresponding to the top or bottom panels respectively. Images depict TdTomato reporter (red) or endothelial-specific CD31 (green).
